# Parvovirus B19 and Cellular Transcriptome Dynamics in UT7/EpoS1 Cells

**DOI:** 10.3390/v18090988

**Published:** 2026-09-08

**Authors:** Niccolò Guglietta, Federica Bichicchi, Ilaria Gasperini, Elisabetta Manaresi, Giorgio Gallinella

**Affiliations:** 1 Department of Pharmacy and Biotechnology, University of Bologna, 40138 Bologna, Italy; niccolo.guglietta2@unibo.it (N.G.); federica.bichicchi2@unibo.it (F.B.); ilaria.gasperini3@unibo.it (I.G.); elisabetta.manaresi@unibo.it (E.M.); 2 Microbiology Unit, IRCCS Azienda Ospedaliero-Universitaria di Bologna, 40138 Bologna, Italy

**Keywords:** Parvovirus B19, UT7/EpoS1 cells, high-throughput sequencing, bioinformatics, transcriptome analysis, interaction networks

## Abstract

Parvovirus B19 (B19V) is a human ssDNA virus with ample pathogenic potential, characterized by a selective tropism for erythroid progenitor cells (EPCs) in the bone marrow. In vitro, in addition to EPCs, UT7/EpoS1 cells are widely used as a model cell system, permissive to viral replication, although in a restrictive pattern. In our work, we applied mRNA high-throughput sequencing technology (HTS) and a dedicated bioinformatic pipeline to investigate both viral and cellular expression profiles in the course of B19V infection of UT7/EpoS1 cells. Mapping of the viral transcriptome detailed the differential expression pattern across early and late time points in the course of infection, at 2, 16 and 48 h post-infection (hpi). Analysis of the cellular transcriptome indicated that downregulation of genes involved in the immune/cytokine/interleukin response was prominent from earlier time points throughout the time course of infection. Upregulation of genes involved in cell stress response was found at 2 hpi, and genes involved in cell cycle regulation were affected mainly at 16 hpi and 48 hpi. A comparative analysis was performed with EPCs, showing similarity in their viral expression profile but substantial divergence in the virus-induced dysregulation of the cellular transcription pattern. This dual-transcriptome analysis of infected UT7/EpoS1 cells and comparison with EPCs provides groundwork for future research aimed at providing a better definition of the pathogenic mechanisms of B19V.

## 1. Introduction

Parvovirus B19 (B19V), a member of the *Erythroparvovirus* genus in the *Parvoviridae* family [1], is a widely distributed human virus with ample pathogenic potential. B19V is characterized by a selective tropism for progenitor cells in the erythroid lineage (EPC), with strict dependence on specific lineage commitment, the differentiation stage and the proliferation rate of this cell population. This restriction is the result of a combination of cell susceptibility, linked to the presence on the cell membrane of specific receptor moieties for B19V virions, and permissiveness, linked to erythroid-specific intracellular signaling pathways. In EPCs, B19V exerts a cytotoxic effect, with consequent blockade of erythropoiesis, leading to typical pathologies of the hematopoietic compartment, such as erythroid aplastic crises and pure erythrocyte aplasia. B19V can infect other cell types, notably endothelial or connective tissue cells; in these, internalization may not depend on receptor interaction, the replicative cycle is not normally productive, and consequences are related to the induction of an inflammatory response [2,3].

Studies on B19V have been hampered by its demanding in vitro growth requirements. Apart from primary erythroid progenitor cell (EPC) cultures, differentiated from peripheral blood mononuclear cells [4,5], few cell lines are permissive for B19V and can be useful as a model system for studying virus–cell interactions. Originally derived from the human leukemic cell line UT7, UT7/Epo cells are a subclone selected for differentiation towards an erythroid phenotype and are strictly dependent on erythropoietin (Epo) for growth and survival [6]. UT7/Epo cells [7], and, in particular, the derived subclone UT7/EpoS1 [8,9], are most commonly used for in vitro studies of the B19V lifecycle. Compared with the parental cells, UT7/EpoS1 cells show a higher permissiveness to B19V, although still allowing only a restricted pattern of infection and limited support for viral replication [10,11]. Understanding the cell-type-specific determinants of restriction of viral replication and the specific impact of the virus on the cell expression profile are topics of relevance.

**Figure 1:**
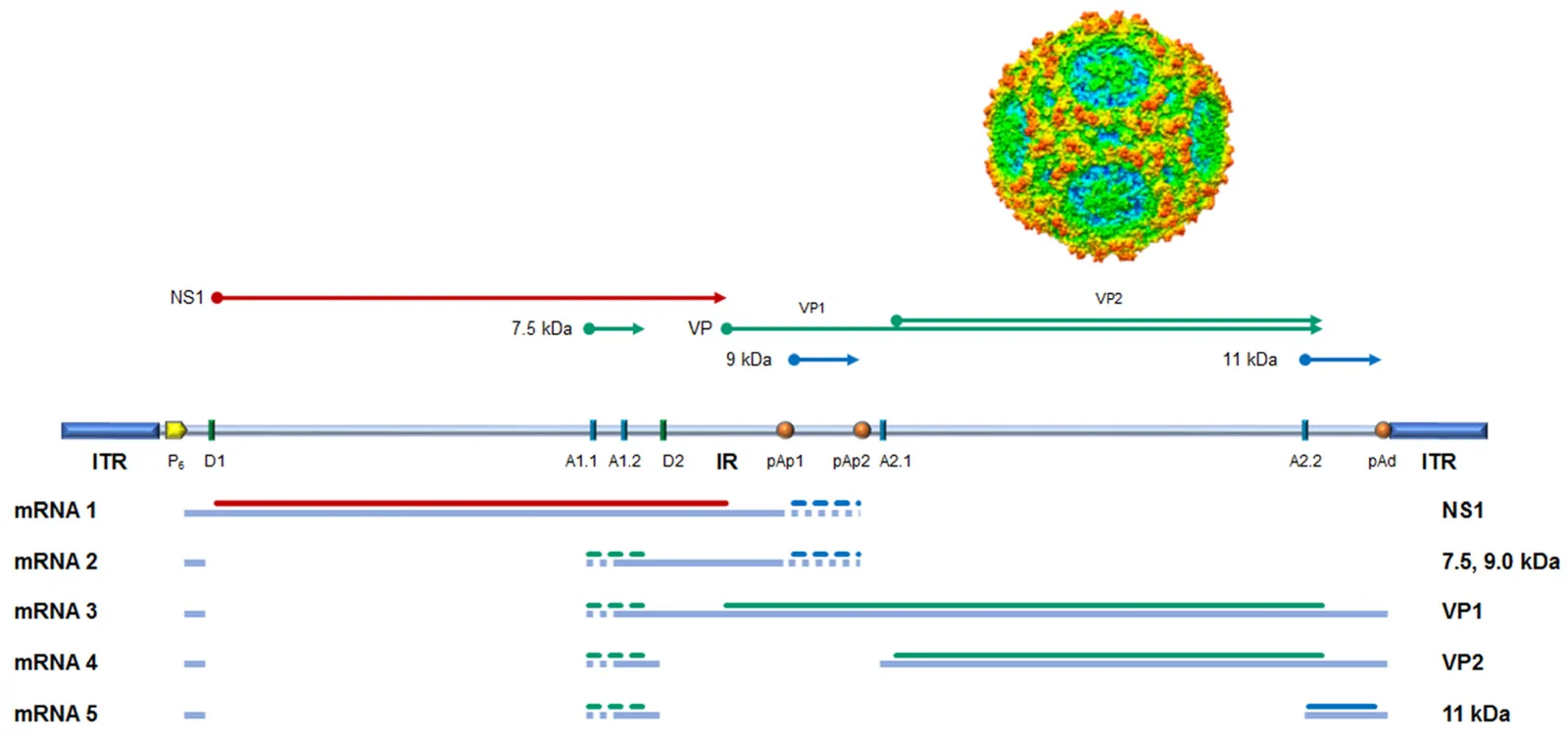
B19V genome organization, transcription map, encoded proteins and capsid structure. Diagram of B19V genome (inverted terminal region, ITR; internal region, IR) and cis-acting functional sites (P6, promoter; pAp1 and pAp2, proximal cleavage–polyadenylation sites; pAd, distal cleavage–polyadenylation site; D1 and D2, splice donor sites; A1.1, A1.2, A2.1, and A2.2, splice acceptor sites). Bottom: Simplified transcription map of the B19V genome, indicating the five classes of mRNAs (mRNA 1–5) with respective alternative splicing/cleavage forms (dashed) and their coding potential. Top: Coding sequences for the viral proteins. NS1, non-structural protein NS1; VP, structural proteins, collinear VP1 and VP2, assembled in a T = 1 icosahedral capsid; and 7.5 kDa, 9.0 kDa, and 11 kDa: minor non-structural proteins. Figure from [12]. Capsid structure from [13].

High-throughput sequencing (HTS) techniques are powerful tools for thorough investigation of genomic and transcriptomic layers in biological systems, such as virus–cell systems. In particular, mRNAseq techniques can be used in a dual-targeting approach to trace both the dynamics of the viral transcriptome (outlined in Figure 1 for B19V) and the modification of the host–cell transcriptome as a response to virus-induced stress. By these means, we previously developed a dedicated experimental and bioinformatics pipeline to investigate the B19V-EPCs system [12]; we now applied such an experimental workflow to investigate the viral expression profile and the induced effects on the host cell expression profile in B19V-infected UT7/EpoS1 cells. A focus on UT7/EpoS1 cells is justified by their relevance as a standard cellular model, commonly used for investigation of the B19V lifecycle. In comparison with primary EPC cultures, characterized by population heterogeneity and ongoing differentiation [5,12], UT7/EpoS1 cells constitute a more homogeneous cell population, offering relevant comparison terms for appraisal of variation in viral and virus-induced cellular expression profiles.

## 2. Materials and Methods

### 2.1. Cell Characterization

**Cells**: UT7/EpoS1 cells, originally established as described in [8,9], were obtained from S. Wong and K. Brown, Hematology Branch, National Heart, Lung and Blood Institute, Bethesda, MD, USA [10]. The cells were cultured in IMDM supplemented with 10% FBS and 2 U/mL Epo and maintained at 37 °C and 5% CO_2_ at a maximal density of 10^6^ cells/mL.

**Cytofluorimetric Analysis**: Aliquots of 5 × 10^5^ cells were stained with antibodies specific for globoside and CD71 and via VP1u binding for the VP1u receptor. Globoside expression was evaluated by rabbit anti-globoside polyclonal antibodies (Matreya, Cayman Chemical, Ann Arbor, MI, USA), followed by anti-rabbit- FITC (DakoCytomation, Glostrup, Denmark). CD71 expression was evaluated by phycoerythrin (PE)-labeled monoclonal antibodies (BD Biosciences, San Jose, CA, USA). VP1u receptor (VP1uR) expression was evaluated by binding of recombinant VP1u-FLAG (kindly provided by C. Ros, Bern, Switzerland [14]) followed by rabbit anti-FLAG (Agilent Technologies, Santa Clara, CA, USA) and secondary goat anti-rabbit Dylight488 (Immunoreagents, Raleigh, NC, USA). Data were acquired by FACSCalibur and were analyzed using the Cell Quest Pro software ver. 6.0 (both Becton Dickinson, Franklin Lakes, NJ, USA).

### 2.2. Infection and Sampling

**Virus**: B19V was obtained from a cloned synthetic genome, as described in [15]. For infection, UT7/EpoS1 cells were incubated at a density of 10^7^ cell/mL, in the presence of B19V to a multiplicity of infection (moi, expressed as geq/cell) of 10^3^ geq/cell, for 2 h at 37 °C. After removal of the inoculum virus, the cells were incubated at 37 °C in 5% CO_2_ in complete growth medium at an initial density of 10^6^ cells/mL.

**Sampling and nucleic acid purification:** Sampling was carried out in triplicate experimental series for uninfected UT7/EpoS1 cells as controls, labeled as 0-hpi, and for infected UT7/EpoS1 cells at 2-, 16- and 48-hpi (hours post-infection). For each sample, equal amounts of cell cultures, corresponding to 1.5 × 10^5^ cells, were collected and further processed for molecular analysis and mRNAseq.

### 2.3. Quantitative Molecular Analysis

**qPCR and qRT-PCR**: Pelleted cells were then processed by the Maxwell Viral Total Nucleic Acid kit on a Maxwell MDx platform (Promega, Madison, WI, USA) to obtain a purified total nucleic acid fraction in elution volumes of 150 µL. A quantitative evaluation of target nucleic acids was carried out by qPCR assays in a Rotor-Q system (Qiagen, Hilden, Germany). For the analysis of B19V DNA, aliquots of the eluted nucleic acids (corresponding to ~500 cells) were directly amplified in a qPCR assay (Maxima SYBR Green qPCR Master Mix, Thermo Fisher Scientific, Carlsbad, CA, USA). For the analysis of B19V RNA, parallel aliquots were first treated with the Turbo DNAfree reagent (Ambion, Kaufungen, Germany) before amplification in a qRT-PCR assay (Express One-step SYBR GreenER Kit, Invitrogen, Carlsbad, CA, USA). Standard cycling programs were used, followed by a melting curve analysis to define the Tm of amplified products. Primer pairs (Appendix A Table A1) were selected to allow quantitation of viral DNA, total viral RNA, and selected subsets of viral RNA. Quantitative evaluation of the target was obtained by absolute quantitation on external calibration curves, as described in [16,17].

**Flow-FISH:** For each sample, equal amounts of cell cultures, corresponding to 1.5 × 10^6^ cells, were collected and processed by flow-FISH assay for the detection of viral nucleic acids, as described in [18]. Cells were fixed in PBS–paraformaldehyde (0.5%) at 4 °C, permeabilized in PBS containing 0.2% saponin, and resuspended in 50 μL of a hybridization solution containing 70% formamide. A digoxigenin-labeled DNA probe mixture specific for B19V nucleic acids was generated by the random-priming method on the cloned full-length genomic DNA template (Dig-High Prime, Roche, Basel, Switzerland). The probe mixture was separately denatured at 95 °C for 5 min and then added at 400 ng/mL to the cell suspension, previously heated at 70 °C for 5 min. Hybridization occurred at 37 °C for 12 h; cells were then washed at RT for 15 min in 1× Stringent Wash (Zytovision, Bremerhaven, Germany). Finally, cells were incubated with an FITC-conjugated anti-digoxigenin antibody (Roche), washed twice in PBS, and resuspended in PBS for flow cytometry analysis.

### 2.4. mRNAseq Analysis

**Sample preparation**: Collected samples were processed using the Maxwell 16 SimplyRNA Cells Kit on a Maxwell MDx platform (Promega) to obtain an RNA fraction in elution volumes of 50 µL. The amount of purified RNA was determined with a Qubit 4 Fluorometer (Invitrogen) using a Qubit RNA BR (Broad Range) Assay Kit. RNA integrity was assessed by the Bioanalyzer RNA assay (Agilent technologies) before HTS mRNA sequencing.

**Sequencing**: mRNAseq was carried out by IGA Technology Services (Udine, Italy). Libraries were prepared using a CORALL Total RNA-Seq Library Prep Kit and a RiboCop rRNA Depletion Kit (both Lexogen, Vienna, Austria) to remove ribosomal RNA from the samples. Final libraries were checked with both a Qubit 2.0 Fluorometer and an Agilent Bioanalyzer DNA assay. Sequencing was performed in paired-end 150 bp mode on NovaSeq6000 (Illumina, San Diego, CA, USA). Raw data containing the base calls were demultiplexed and converted to FASTQ files using Illumina’s Bcl2Fastq 2.20 software, and adapter sequences were masked with Cutadapt v1.11.

### 2.5. Data Analysis

**Read Trimming**: Trim Galore! (v0.6.10) was used in paired-end mode to remove the adapters and trim off the low-quality bases found at the ends of each read. FastQC (v0.12.1) was utilized to check the quality scores of the samples before and after trimming.

**Read Mapping**: Trimmed reads were aligned to the synthetic B19V EC Genotype I consensus sequence (GenBank KY940273.1) with HISAT2 (v2.2.1) [19]. Aligned reads were then converted to BAM files using SAMtools (v1.22) [20] before sorting and indexing.

**Feature Mapping**: Reads mapping to splice and cleavage–polyadenylation sites were extracted by string search using seqkit (v2.10.0) [21] (Appendix A, Table A2). Captured expression signals were collected into FASTQ files and later aligned to the B19V EC Genotype I consensus sequence (GenBank KY940273.1) with HISAT2. Sorted and indexed BAM files were used to generate coverage graphs with a custom Python script leveraging the *pysam* library (v0.23.3) together with *matplotlib* (v3.10.5) to generate the graphs.

**Transcript Count**: Salmon (v1.10.3) [22] was used to quantify the transcript abundance from the trimmed FASTQ files. The GENCODE (release 48) [23] dataset was utilized. The quantified counts were then imported into the R statistical environment (v4.5.1) using the *tximport* R package (v1.36.0) [24] and the metadata file of the transcriptome containing the translation from transcript identifier to gene symbol. Around 22% of all transcript identifiers lacked an associated gene symbol; the affected transcripts were, thus, excluded from the downstream analysis.

**Statistical Design:** A multi-factorial linear model was designed, incorporating the variables of hours post-infection (hpi) and infection status, along with their interaction terms.

Within *edgeR*’s framework [25], the expected read count $\mu_{gi}$ for gene *g* in sample *i* is modeled as a log-linear model of the form

(1) $$\log\mu_{gi}=x_{i}^{T}\beta_{g}+\log L_{i}$$

where $x_{i}$ is a covariate vector encoding the experimental design for sample *i*, $\beta_{g}$ is a vector of coefficients representing the experimental effects and log-fold-changes, and $L_{i}$ is the effective library size for sample *i*.

The observed read count $y_{gi}$ is assumed to follow a mixture distribution across biological replicates whose variance relates to the mean quadratically:

(2) $$var\left(y_{gi}\right)=\sigma_{g}^{2}\mu_{gi}+\psi_{g}\mu_{gi}^{2}$$

where $\sigma_{g}^{2}$ represents the technical variability, or quasi-dispersion, and $\psi_{g}$ captures biological variability, commonly referred to in the field as the biological coefficient of variation (BCV) [26]. Tested contrasts, which are linear combinations of the statistical model coefficients, were defined in order to perform comprehensive tests for gene dysregulation. Design matrix and contrast creation were performed using the *limma* R package (v3.64.1) [27].

**Differential Expression Testing and Modeling**: Differential gene expression analysis (DGEA) was carried out in the R environment using the *edgeR* (v4.6.2) package [28]. The gene-level count matrix was first imported into a *DGEList* object, which encapsulates the raw count data as well as the sample grouping information and the experimental design matrix. Genes with a low expression level across all samples were removed to reduce the background noise in the data. Library size normalization was carried out using the trimmed mean of M-values (TMM).

**Exploratory Data Analysis:** Principal component analysis (PCA) was performed on the log-transformed counts per million (logCPM) gene counts and visualized using the *factoextra* R package (v1.0.7). Multidimensional scaling (MDS) was applied and visualized with the *plotMDS* function of *edgeR* in order to assess the expression profiles of the different samples and replicates.

**Model Fitting:** The BCV of each gene was computed and compared with its average log-transformed CPM value with the *plotBCV* function of the *edgeR* package. The robust quasi-likelihood negative binomial generalized log-linear model from *edgeR* was trained using the *glmQLFit* function in order to determine profiles of differential gene expression. Subsequently, the quasi-likelihood dispersion of the model was plotted using the *plotQLDisp* function to diagnose whether the model was able to describe the data correctly or not.

**Contrast Testing:** Differential gene expression in different conditions was tested by evaluating individual contrasts under the conditions of (i) no log-fold change (logFC) threshold, employing *glmQLFTest*, and (ii) with a logFC threshold of 1.6 using *glmTreat*. The results from both methods were processed with *decideTests*, a function within *edgeR*, which classifies genes as upregulated, downregulated or not significantly dysregulated; a *p*-value < 0.05 was used as the threshold for significance. The default Benjamini–Hochberg (FDR) procedure was employed to correct for multiple testing. Visualizations were created in the form of volcano plots using the *ggplot2* R package (v3.5.2) and in the form of heatmaps and UpSet plot visualizations using the *ComplexHeatmap* R package (v2.24.0) [29].

**Gene Set Enrichment Analysis:** Competitive gene set testing was performed using the Camera method, as implemented in the *edgeR* package [30], to evaluate the dysregulation of biological pathways found in the MSigDB hallmark collection [31] whilst accounting for inter-gene correlation. Lollipop plots were created with *ggplot2*.

## 3. Results

### 3.1. Course of Infection of B19V in UT7/EpoS1 Cells

**Figure 2:**
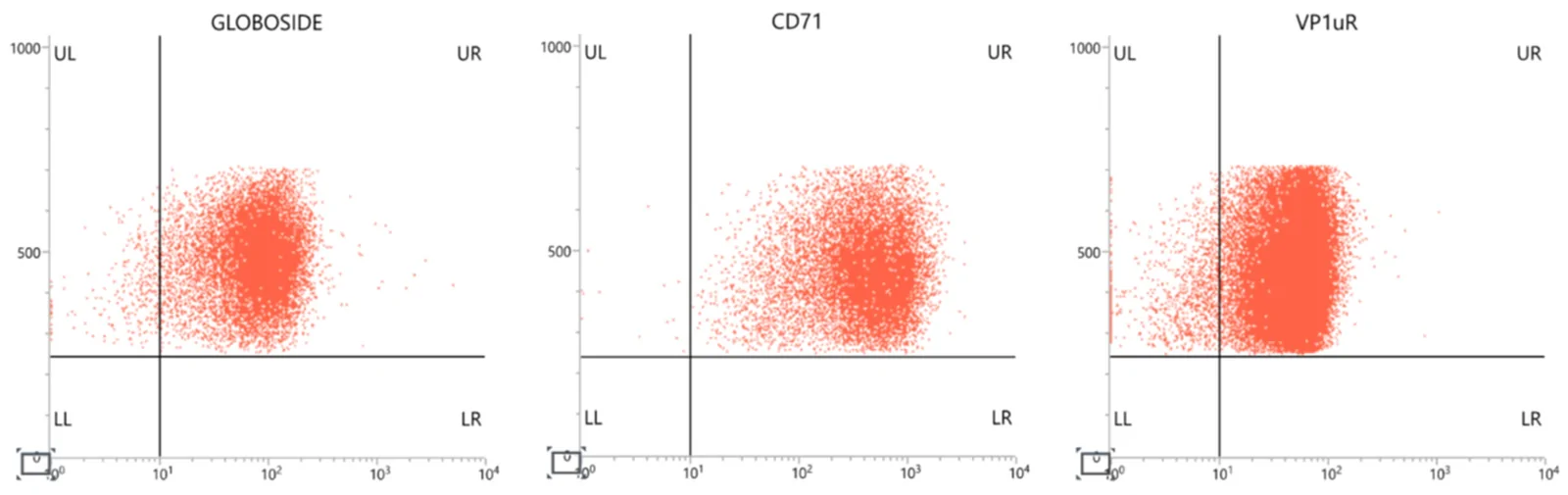
Phenotypical characterization of UT7/EpoS1 cells. Staining for globoside, CD71, and binding of VP1u (fluorescent labeling, X-axis; FSC, Y-axis). Positive cells are in UR quadrant and constitute 98.1%, 99.9%, and 96.9% of the cell population, respectively.

UT7/EpoS1 cells were first characterized by flow cytometry to assess the presence and distribution of cell surface markers relevant to erythroid differentiation and susceptibility to B19V infection. In particular, the glycolipid globoside, CD71 (hTFR1), and the VP1u ligand were detected and found uniformly present in the whole-cell population (Figure 2).

**Figure 3:**
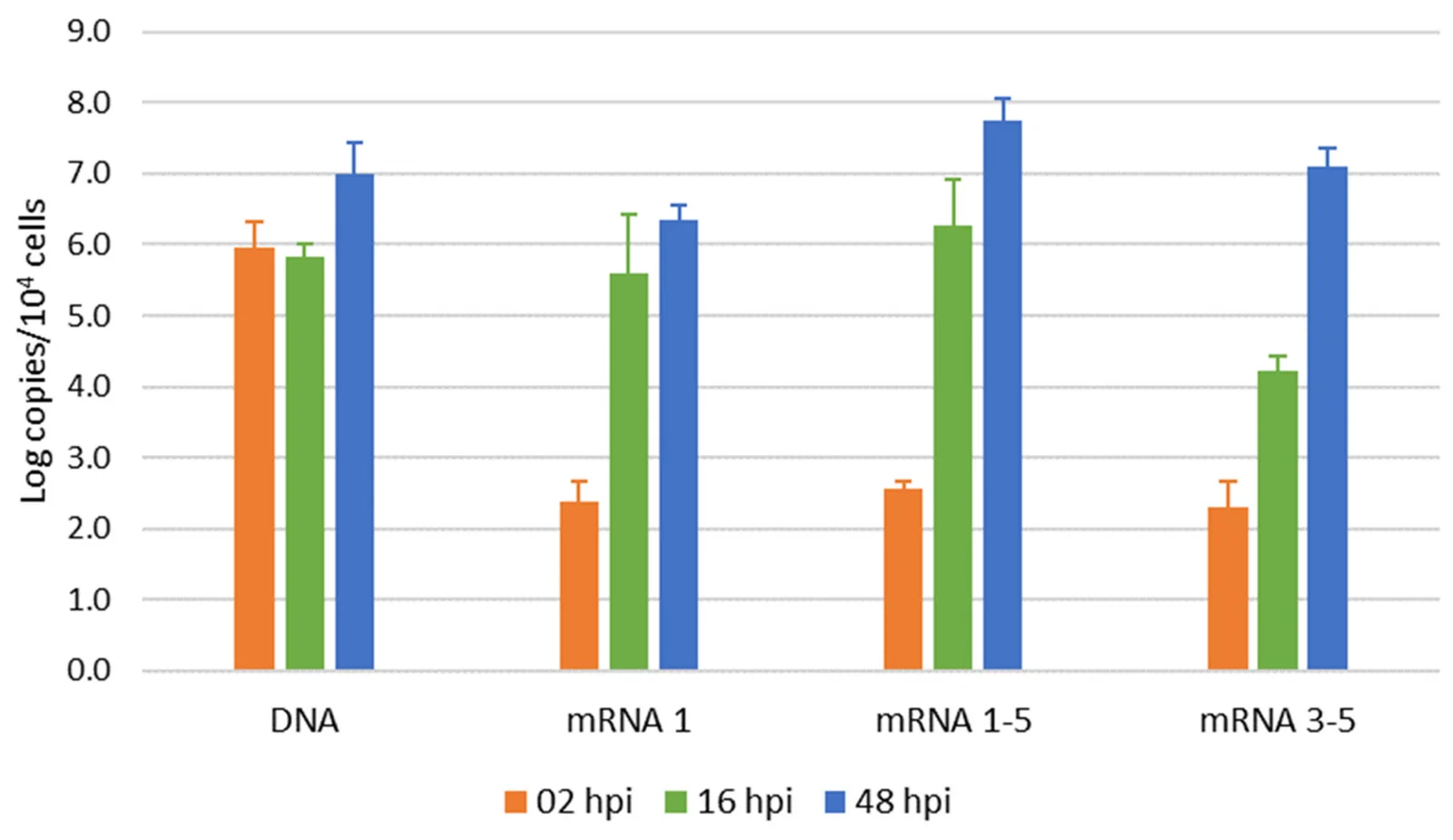
Quantitative analysis of viral genomic DNA, total viral mRNA (mRNA1–5), and mRNA subsets (mRNA1 and mRNA3–5) in EPCs at the indicated time points. Quantitative data obtained from three independent replicate experiments, with each determination in duplicate; bars indicate means + SD.

The course of B19V infection in UT7/EpoS1 cells was monitored by qPCR to quantify variation in the amount of viral DNA and by qRT-PCR to quantify variation in the amount of total (mRNA1–5) and relevant subsets of viral mRNAs, in particular, the proximally cleaved, unspliced mRNAs (mRNA1) or the distally cleaved, spliced mRNAs (mRNA3–5). The amount of viral DNA detected in the cell population remained stable from 2 to 16 hpi and showed a net increase of 1.2 log from 16 to 48 hpi, confirming the productive although restricted infection pattern in UT7/EpoS1 cells. Total viral mRNA, barely detectable at 2 hpi, showed increasing amounts at 16 and 48 hpi, also confirming the productive pattern of infection (Figure 3). Comparing the abundance of mRNA subsets between these 16 and 48 hpi time points, a relatively higher abundance of proximally cleaved unspliced transcripts, coding for the NS1 protein, was found at 16 hpi (20.6% and 0.9% respectively). On the contrary, a relatively higher amount of distally cleaved spliced transcripts, coding for VP1/2 and 11 kDa protein, was found at 48 hpi (4.0% and 22.4% respectively). Therefore, the known biphasic early/late expression pattern of B19V expression was confirmed in the presented experimental setting, constituting a framework for interpretation of mRNAseq data.

**Figure 4:**
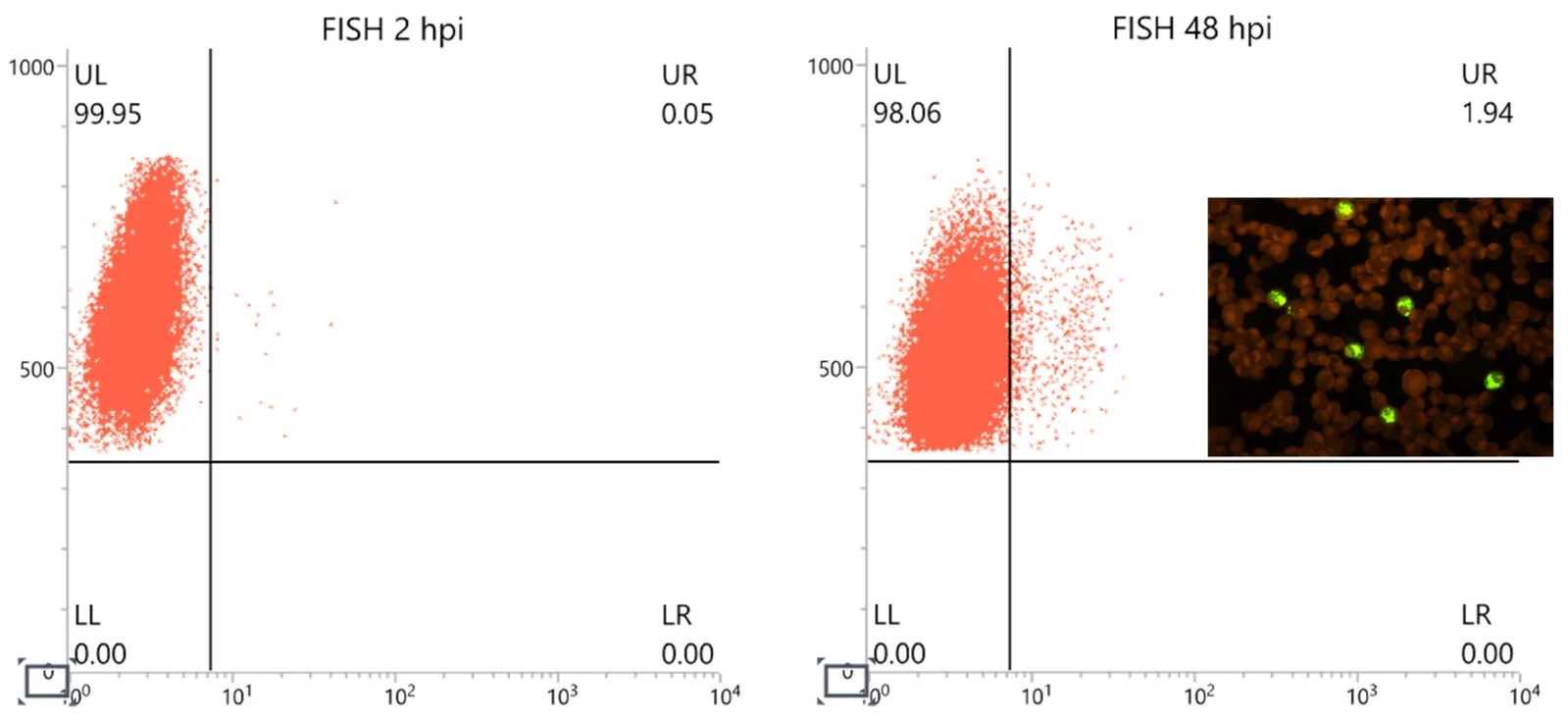
Flow-FISH for viral nucleic acids in control and infected UT7/EpoS1 cells at 2 and 48 hpi; FITC labeling, X-axis. FSC, Y-axis. FISH-positive cells (inlet) are in UR quadrant.

The outcome of infection in the cell population was further confirmed by FISH detection of viral nucleic acids at 48 hpi. The fraction of positive, productively infected cells, at the experimental moi of 10^3^ virus/cell, was evaluated at about 2%, an expected low value in accordance with the known restrictive pattern of infection in UT7/EpoS1 cells (Figure 4) [18]. Therefore, subsequent interpretation of mRNAseq data, at least for viral mRNAs, should be considered relative to such a subset of cells.

### 3.2. mRNAseq Analysis

For the mRNAseq experiments, the analyzed samples included uninfected UT7/EpoS1 cells as a baseline control at 0 hpi and B19V-infected cells collected at 2, 16, and 48 hpi. Triplicate experimental series were processed for RNA purification, and mRNAseq analysis was conducted on about 1 µg of RNA per sample. The rough output yielded 2.9–3.5 × 10^7^ reads per sample.

Read mapping was first conducted on the synthetic B19V reference genome (GenBank KY940273.1). No reads from the uninfected control samples mapped to the B19V genome, as expected. Less than 10^2^ reads per sample were mapped to B19V at 2 hpi; 3.1–3.5 × 10^4^ reads mapped to the B19 genome at 16 hpi (0.01% of total); and 1.3–1.4 × 10^6^ reads mapped to B19V at 48 hpi (0.53% of total). For each sample, mapping of residual reads to the reference human genome (build GRCh38.p14) returned between 69 and 80% correctly mapped reads.

### 3.3. Viral Transcriptome

**Figure 5:**
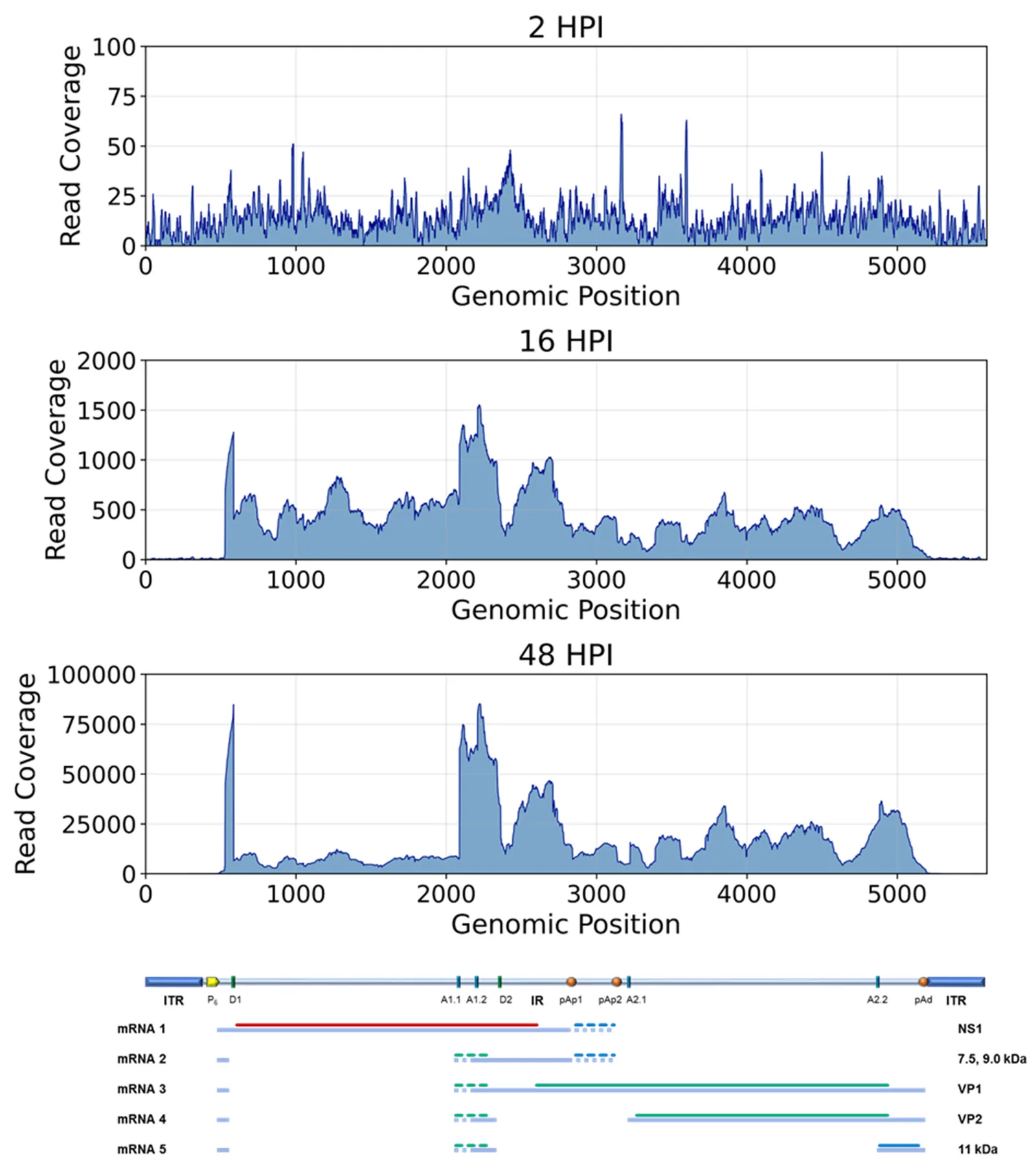
mRNA seq coverage aligned on B19V transcription map (2, 16 and 48 hpi).

Mapping of reads to the B19V genome was visualized using a custom Python script leveraging the *pysam* library together with *matplotlib* (Figure 5).

At 2 hpi, the scattered mapping of very few reads testified to the early onset of viral mRNA synthesis, but did not allow any further characterization of the viral transcriptome. At 16 and 48 hpi, the increasing number of mapped reads allowed for such characterization. Reads were distributed on the genomic template, in different abundances depending on the genomic region and reported inclusion in exons or introns, in different patterns for the different time points. The first increase in read number at 16 hpi correlated with a prevalent mapping to the left-side genomic region, within the proximal cleavage–polyadenylation sites. The substantial increase in reads at 48 hpi correlated with a prevalent splicing of the first intron, coupled to an increased, although irregular, representation of the right-side genomic region, up to the distal cleavage–polyadenylation site (Table 1).

**Figure 6:**
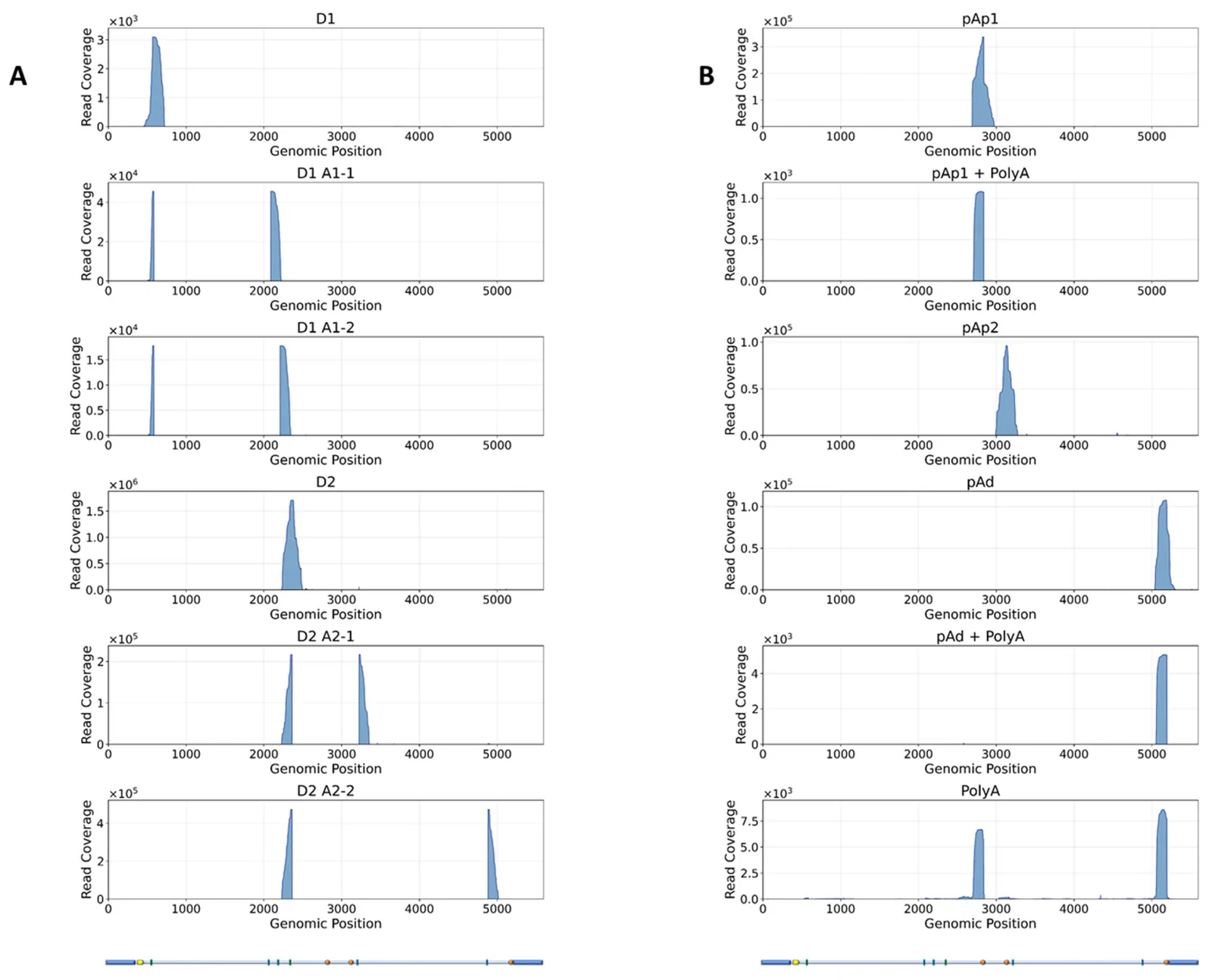
mRNA seq reads mapping to splice junctions (**A**) and cleavage-polyA (**B**) sites at 48 hpi.

A closer inspection was conducted to assess the relevance and pattern of usage of known mRNA processing sites. In detail: the start of transcription at nt. 530; the donor/acceptor splice sites at nt. 586/2089-2209 (D1/A1.1–A1.2); the donor/acceptor splice sites at nt. 2363/3224-4883 (D2/A2.1–A2.2); the pAp1 cleavage–polyadenylation site at nt. 2842; the pAp2 site at nt. 3142, and the pAd distal cleavage–polyadenylation site at nt. 5189. To this purpose, reads spanning reported splice junctions and cleavage–polyadenylation sites were specifically selected and mapped, and their relative abundance was determined (Figure 6).

Considering the splicing process, the first intron showed a prevalent frequency of splicing events, increasing from early to late time points (13% vs. 5% unspliced vs. 87% to 95% spliced transcripts), and usage of proximal to distal acceptor sites. The second intron showed a more balanced and constant frequency of splicing (~40% unspliced transcripts) and usage of proximal and distal acceptor sites. Considering the cleavage-polyA process, usage of the pAp1 site was prevalent at both early and late time points; the pAp2 site was not represented above background; and the pAd site showed increasing frequency at late time points (Table 2).

### 3.4. Cellular Transcriptome

**Figure 7:**
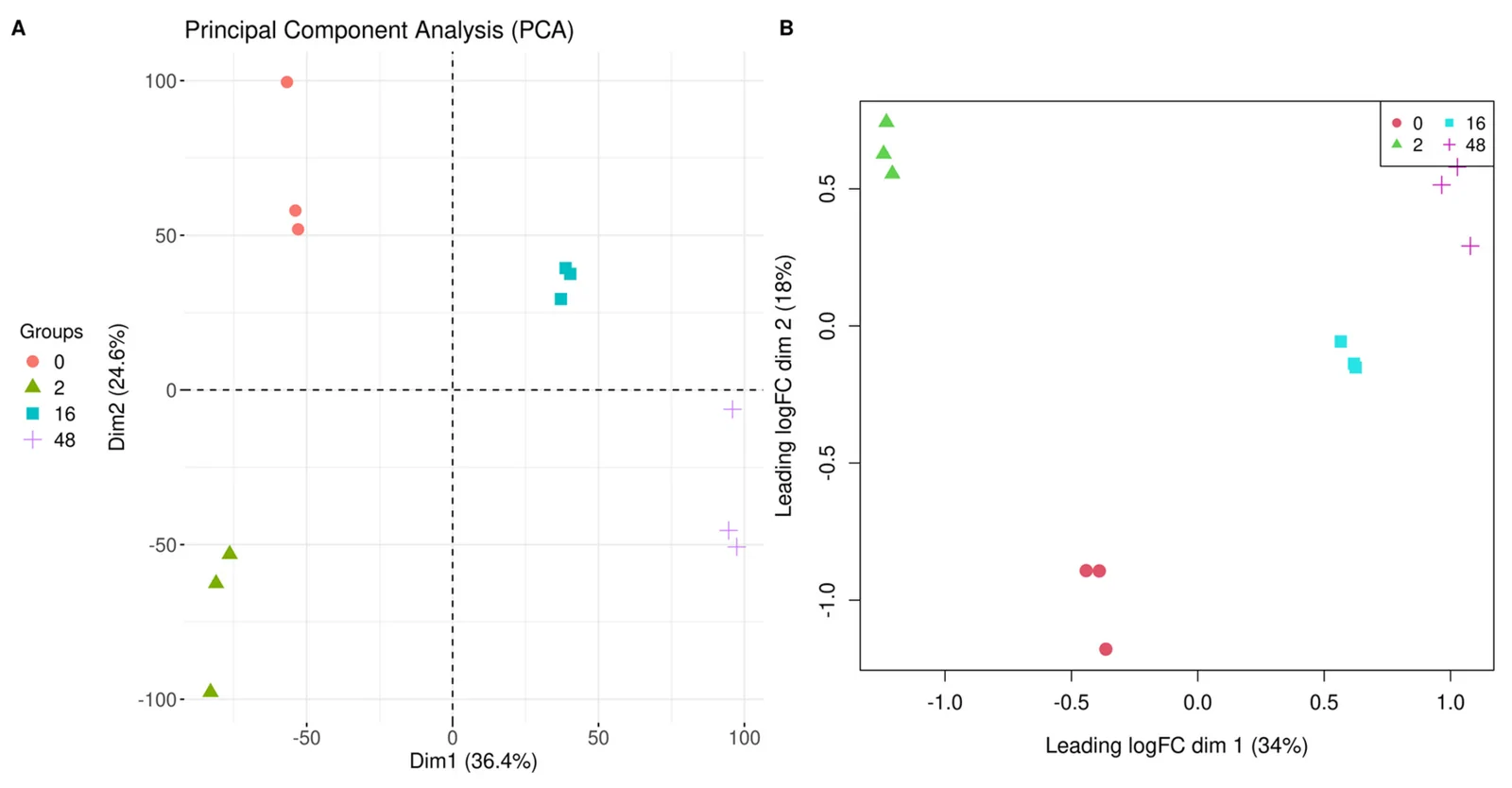
(**A**) PCA plot; the two main dimensions account for 61% of total variation. (**B**) MDS plot; the two main dimensions account for 52% of total variation. In both PCA and MDS plots, the samples cluster according to the hpi variable, while the tight clustering of the replicates with one another certifies the similarity between them.

Variation in the cellular transcriptome was also monitored by comparing uninfected UT7/EpoS1 cells, as a baseline control at 0 hpi, and B19V-infected cells collected at 2, 16, 48 hpi. Principal component analysis (PCA) (Figure 7A), performed on the log-transformed CPM values, and multidimensional scaling (MDS) analysis (Figure 7B) showed significant gene expression differences between sample groups, clearly separating uninfected from infected samples, coupled with low variability within groups.

**Figure 8:**
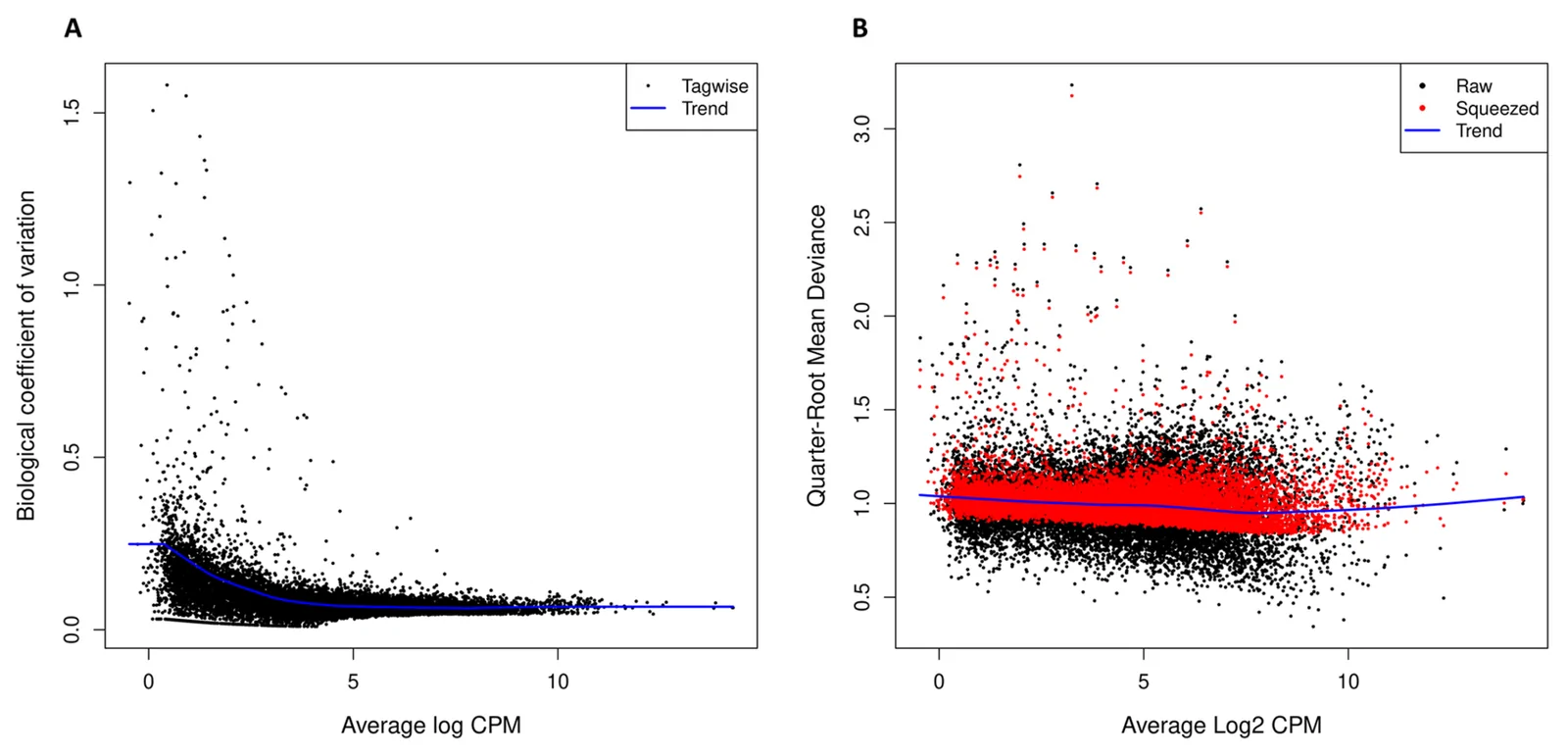
(**A**). BCV plot. For the analyzed samples, values are relatively low and constant, indicating the high statistical power in discerning DEGs in the samples. (**B**). Quarter-root mean deviance plotted against Log2-transformed CPM, showing the quasi-likelihood dispersion of the edgeR model; the tight clustering of the squeezed data points reveals that the model appropriately captures the underlying structure of the data.

Gene count dispersion was assessed with a biological coefficient of variation (BCV) plot (Figure 8A), comparing the BCV with the average log-transformed CPM value of each gene, which confirmed that experimental data provided enough statistical power to distinguish differentially expressed genes (DEGs) with high reliability. In the quarter-root mean deviance plot (Figure 8B), the tight clustering around a relatively smooth trend line indicates that the model was able to estimate the quasi-likelihood dispersion values correctly and that the variance structure was well estimated by the applied statistical model.

**Figure 9:**
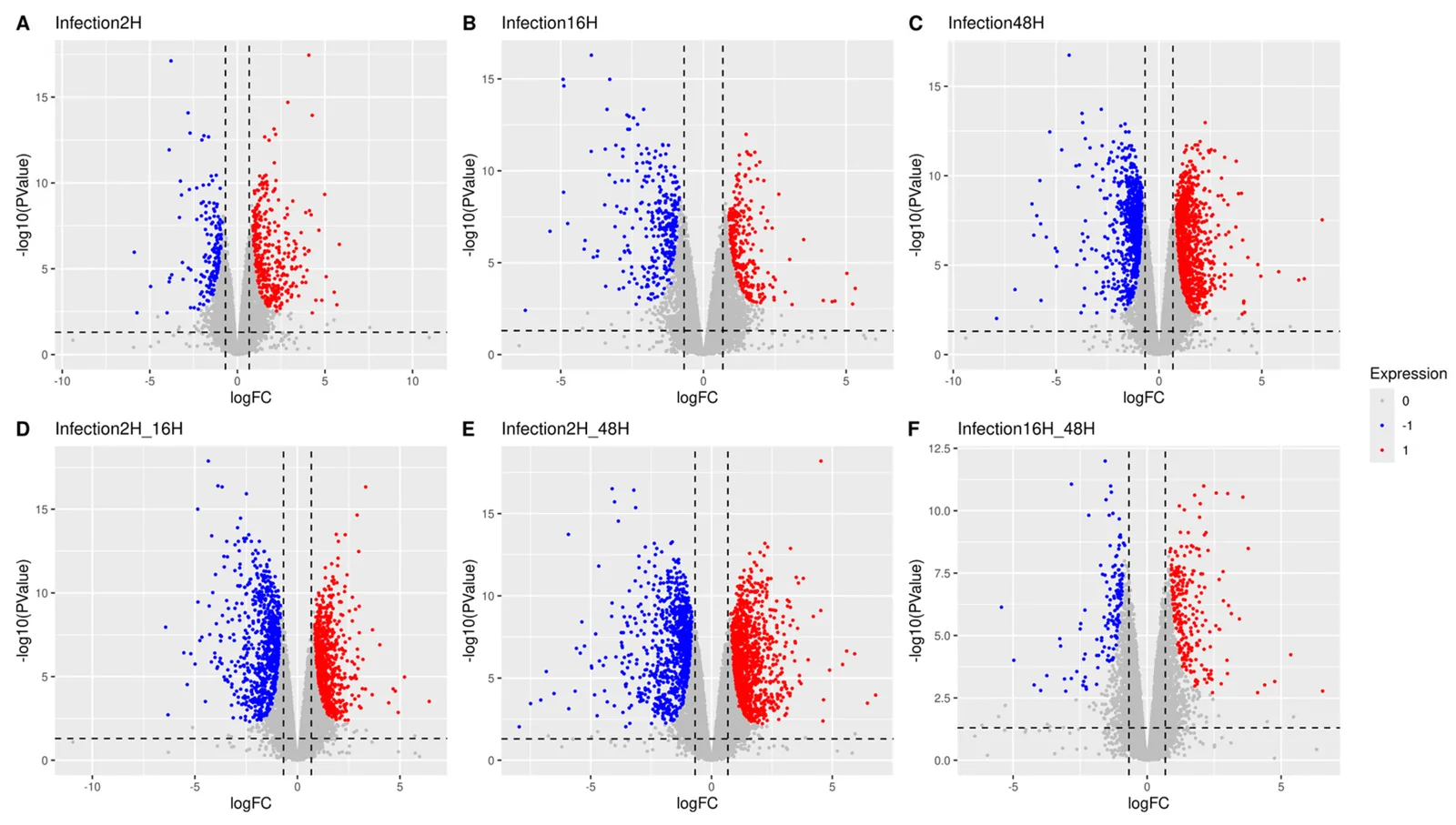
Volcano plots showing the differential gene expression patterns across tested contrasts. (**A**–**C**) compare the different time points post-infection (2, 16 and 48 hpi) with the uninfected cell population (0 hpi). (**D**–**F**) compare the different time points in the infected cell population (2–16 hpi, 2–48 hpi, and 16–48 hpi). On the X-axis, logFC is reported, whilst the Y-axis shows the negative ${log}_{10}$ *p*-values. Each dot on the graph represents a different gene; these are colored in blue for downregulation and in red for upregulation. The different dashed lines represent the thresholds for significance (*p*-value < 0.05) and minimum required logFC (logFC < 1.6 or logFC > 1.6). Gray dots above these thresholds represent non-significantly dysregulated genes. A complete list of differentially expressed genes is in Supplemental File S1.

Differential gene expression analysis (DGEA) identified dysregulated genes across the tested time point contrasts, visualized as volcano plots, comparing the log-transformed fold change of each gene with its negative log-transformed *p*-value (Figure 9). These plots highlight the effects of the progress of infection. Contrasts A-C compare the different time points post-infection (2, 16 and 48 hpi) with the uninfected cell population (0 hpi) and show a progressive increase in gene dysregulation, from the 2 hpi sample to the 48 hpi sample, exhibiting the most differential gene expression. Contrasts D-E compare the different time points in the infected cell population (2–16 hpi, 2–48 hpi, and 16–48 hpi). A substantial difference is observed in the set of differentially expressed genes starting from 2 hpi compared with the 16 and 48 hpi samples, whilst comparison of the 16 hpi with the 48 hpi conditions reveals a minor difference in gene expression, suggesting that most of the virus-induced variation in gene expression is set at early times post-infection.

**Figure 10:**
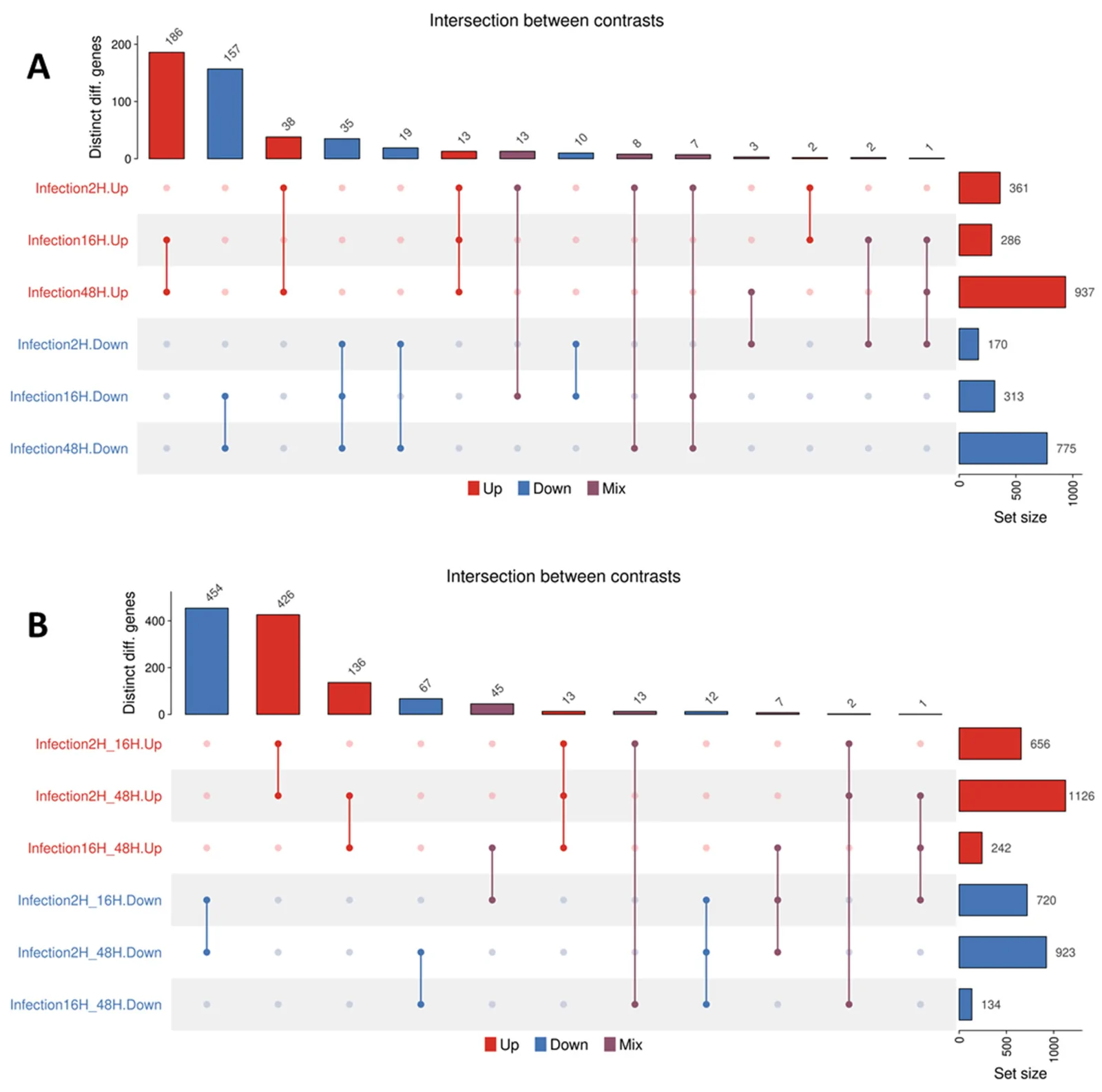
UpSet plots showing the magnitude of sets (rows) and set intersections (columns) of dysregulated genes across tested contrasts. (**A**): different time points post-infection (2, 16 and 48 hpi) compared with the uninfected cell population (0 hpi). (**B**): different time points in the infected cell population (2–16 hpi, 2–48 hpi, and 16–48 hpi).

This scenario is also confirmed by UpSet plots (Figure 10), which visualize the size of the intersections among the sets of differentially expressed genes identified across contrasts. The intersection profiles observed in the infected–uninfected contrast plot (**A**) reveal that 343 genes (~12% of all dysregulated genes) are shared between the 16 hpi and 48 hpi conditions, comprising 186 upregulated and 157 downregulated genes. The next greatest intersection is between the 2 hpi and 48 hpi conditions in both up- and downregulation. Interestingly, 35 genes (~3% of all downregulated genes) are consistently downregulated across all conditions. The intersection in the infected contrasts (**B**) shows that most genes (880 genes, corresponding to ~30% of all dysregulated genes) are common between 2–16 and 2–48 hpi, whilst an additional 203 (~7% of all dysregulated genes) are added between 16 and 48 hpi. The number of genes dysregulated in the contrasting sense is consistently low.

**Figure 11:**
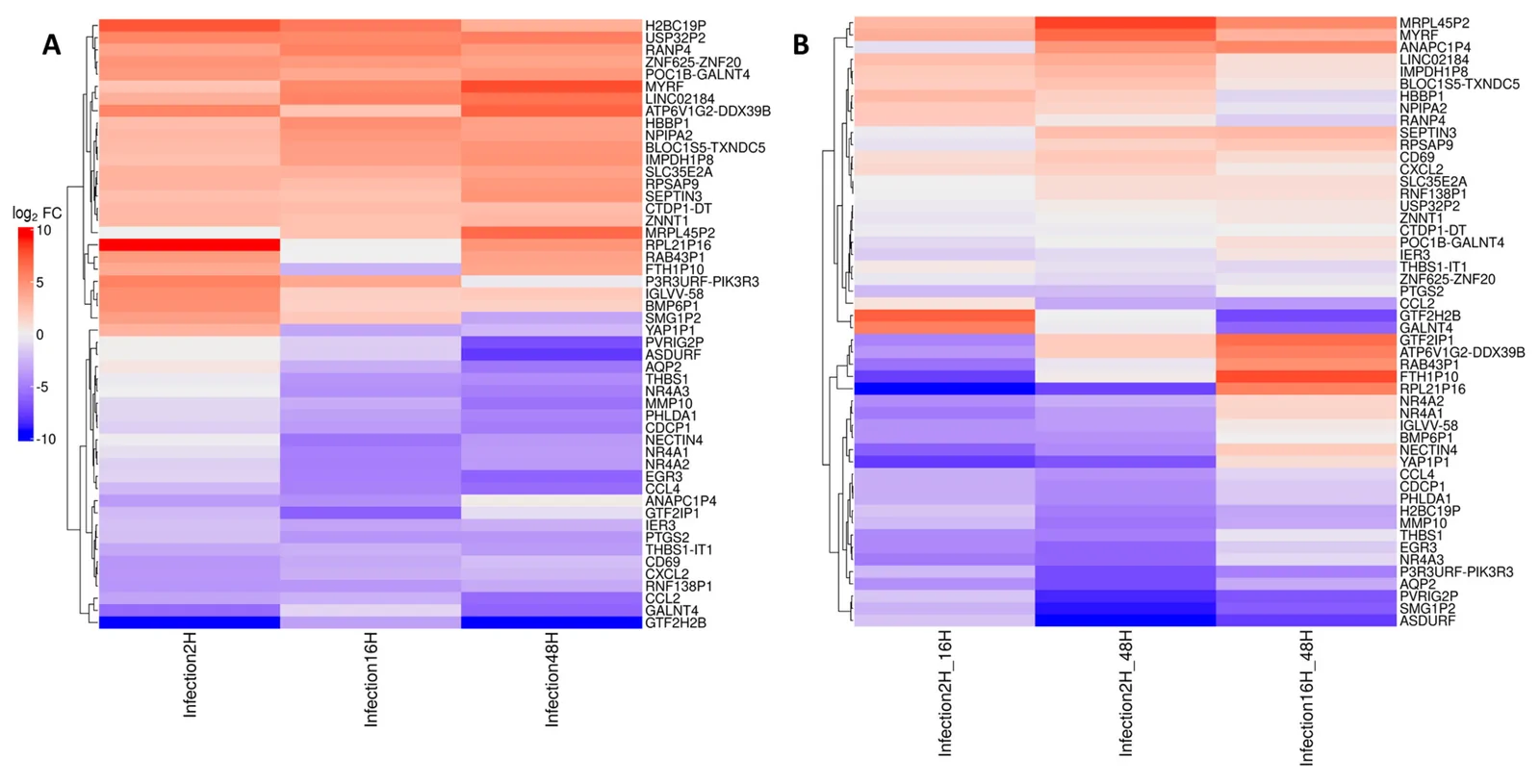
The top-50 DEGs identified across synchronic contrasts are displayed for tested contrasts in a heatmap visualization. (**A**): different time points post-infection compared with the uninfected cell population. (**B**): different time points in the infected cell population. Each cell of the heatmap refers to a specific gene, indicated on the right, and a specific contrast, indicated on the bottom. The dendrogram on the left of each heatmap clusters genes together based on logFC profiles.

To provide insight into gene dysregulation during the course of infection, the fifty most dysregulated genes were visualized in a heatmap representation (Figure 11), in which each row represents a gene, and each column represents a tested contrast. The results indicate a clear separation between two main clusters, either down- or upregulated, for all tested contrasts.

**Figure 12:**
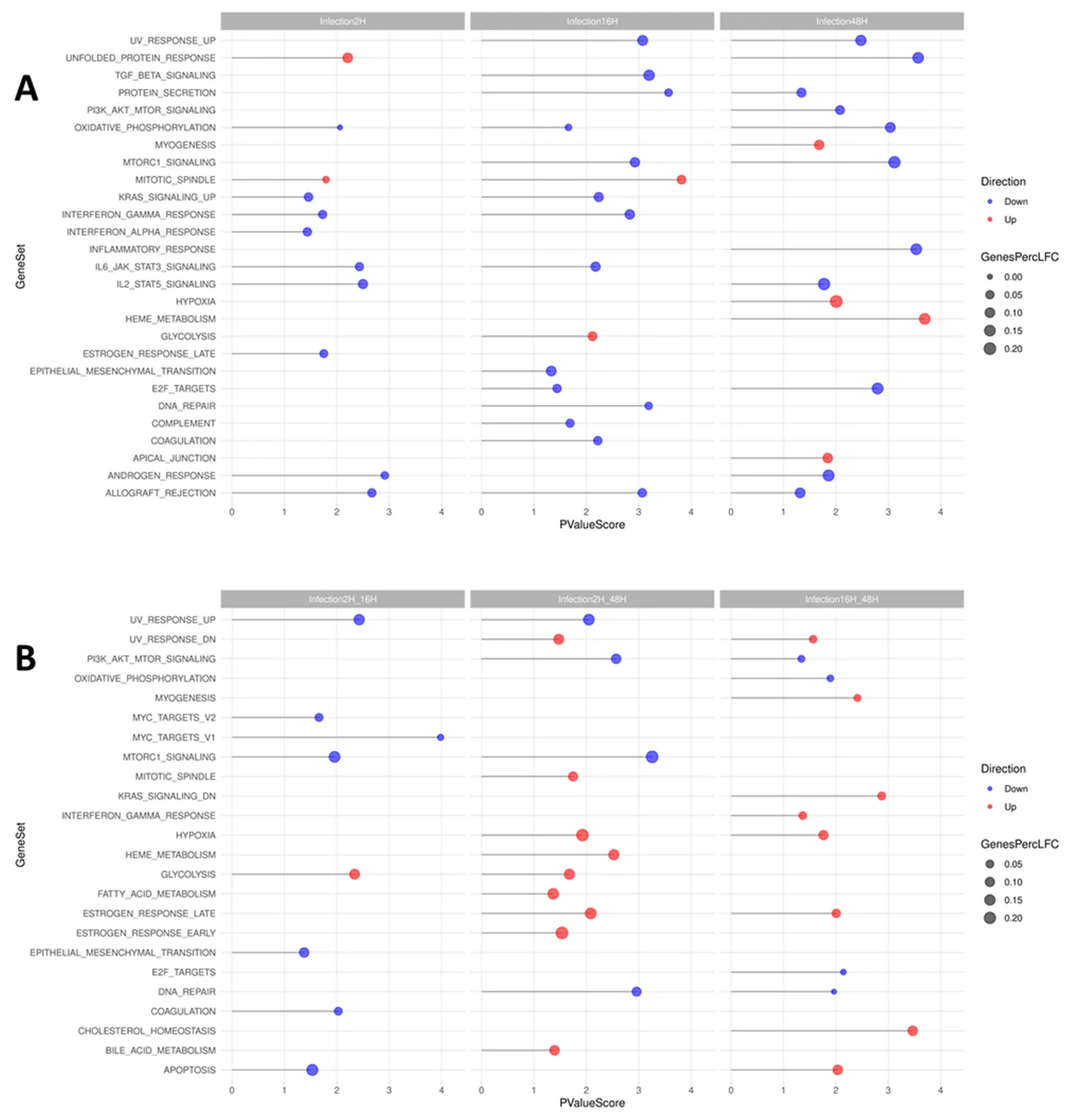
Several pathways of the MSigDB hallmark collection were found to be enriched in the different tested contrasts. (**A**): different time points post-infection compared with the uninfected cell population. (**B**): different time points in the infected cell population. The X-axis reflects the negative ${log}_{10}$ *p*-value, and the size of the dot reflects the percentage of DEGs found in the gene set. The color of the dot reflects either upregulation (red) or downregulation (blue). A complete list of genes is in Supplemental File S2.

Gene set enrichment analysis (GSEA) was carried out on the list of recognized genes using CAMERA (Competitive Gene Set Tests for Digital Gene Expression Data) on the hallmark collection of the Molecular Signature Database (MSigDB) and visualized as a lollipop graph (Figure 12). The top sets were selected based on the enrichment of the set among the different contrasts and their *p*-value scores. For sequential time points–baseline contrasts, GSEA mainly returned downregulation of gene sets, whilst only a few gene sets showed upregulation: at 2 hpi, unfolded protein response; at 16 hpi, mitotic spindle and glycolysis; and at 48 hpi, hypoxia and heme metabolism. Conversely, based on the time point–time point contrasts, more gene sets were upregulated from 2 to 48 hpi, including hypoxia, heme metabolism, glycolysis, estrogen response and bile and fatty acid metabolism.

Finally, within the gene sets previously identified, an exploration of interaction networks was conducted by using the STRING database and a clustered analysis with the Markov cluster algorithm (MCL) [32]. The results are shown in Appendix A, Table A3, and Supplemental File S3 for the most relevant clusters, according to tested contrasts. With reference to the Reactome database [33], the principal pathways involved were as follows: at 2 hpi, immune system signaling by interleukins and cellular responses to stress; at 16 hpi, cytokine signaling, immune system signal transduction, and cell cycle regulation; at 48 hpi, immune system signal transduction and cell cycle regulation; and in the 2–48 hpi time interval, mainly cell cycle regulation and cellular responses to stress.

### 3.5. Cellular Transcriptome: Comparison of UT7/EpoS1 with EPCs

**Figure 13:**
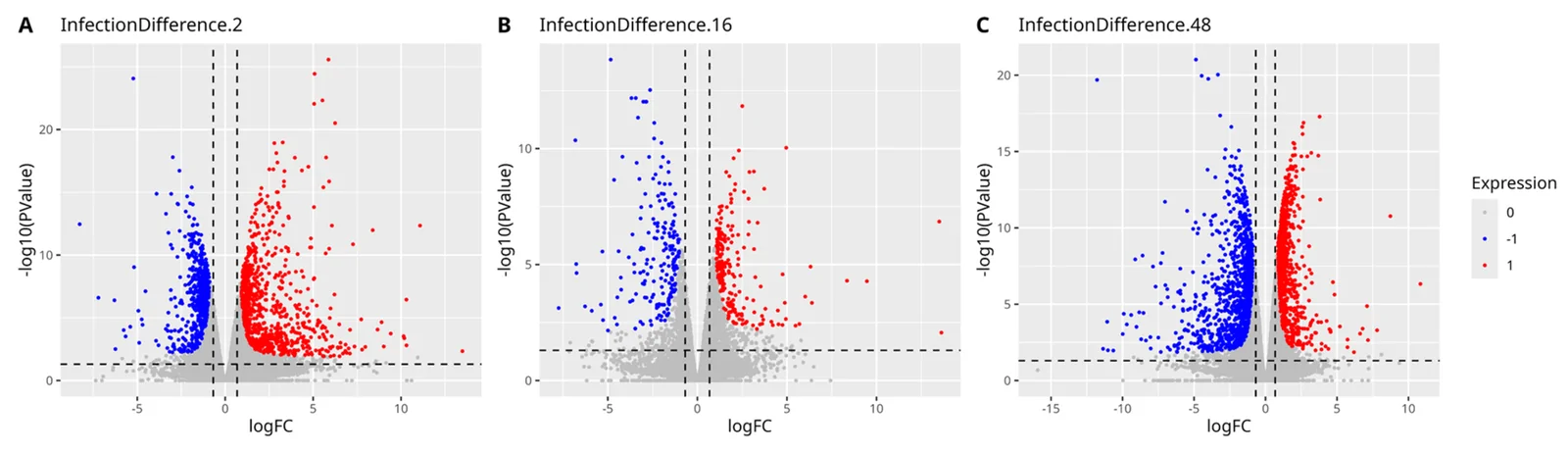
Volcano plots showing the differential gene expression patterns in B19V-infected UT7/EpoS1 compared with EPCs at selected time points ((**A**), 2 hpi; (**B**), 16 hpi, (**C**), 48 hpi). On the X-axis, the logFC is reported, whilst the Y-axis shows the negative ${log}_{10}$ *p*-value. Each dot in the graph represents a different gene; these are colored in blue for downregulation and in red for upregulation. The different dashed lines represent the thresholds for significance (*p*-value < 0.05) and minimum required logFC (logFC < 1.6 or logFC > 1.6). Gray dots above these thresholds represent non-significantly dysregulated genes.

**Figure 14:**
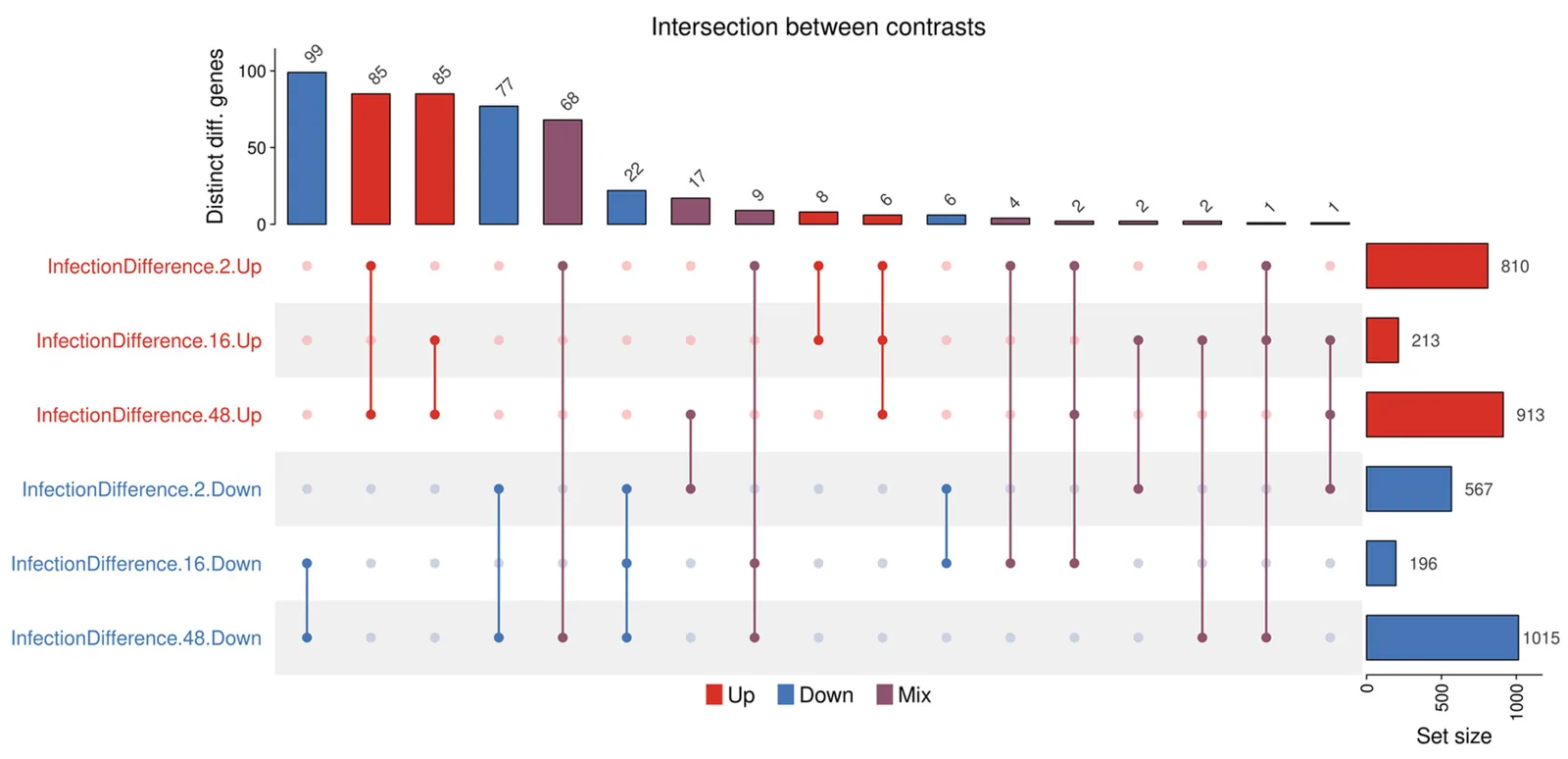
UpSet plot showing the differential gene expression patterns in B19V-infected UT7/EpoS1 compared with EPCs at selected time points.

The direct availability of a transcriptome analysis of B19V-infected erythroid progenitor cells (EPCs) obtained by the same workflow and bioinformatics pipeline [12] allowed for a comparison of the virus-induced expression profile perturbations in these two cellular systems. A simple comparison of UT7/EpoS1 versus EPCs, either uninfected or at the same time points of 2, 16 and 48 hpi, only showed a substantial divergence between the two cellular systems. To elucidate the variations specifically induced by viral infection, the model had to determine the differential variations in the expression profile in the infected and in the basal, not infected, conditions, for both cellular systems (Figure 13). This allowed for individuation of convergent or divergent dysregulated gene sets, eliminating a background of gene sets not significantly affected by the virus (Figure 14).

Comparing the two systems, most genes showed different expression patterns (upset plot, row set size), a result highlighting the difference between the two cell populations. A minor fraction of genes showed concurrent virus-induced dysregulation (upset plot, column set size), and most of these were consistent across the different time points, either upregulated or downregulated in the two systems. GSEA was carried out on MSigDB, followed by exploration of interaction networks by using the STRING database and a clustered analysis with the Markov cluster algorithm (MCL). The results are shown in Appendix A, Table A4, and Supplemental File S4 for the most relevant clusters. With reference to the Reactome database, the principal pathways showing differential regulation between UT7/EpoS1 and EPCs were as follows: at 2 hpi, mitotic G1 phase and G1/S transition and transcription by RNA polymerase II; at 16 hpi, transcription regulator activity, response to cytokine, and interferon signaling; and at 48 hpi, cell cycle, extracellular matrix organization, cytokine signaling, interferon alpha/beta signaling, interleukin signaling, and DNA repair.

## 4. Discussion

Investigation of virus–cell interactions can substantially benefit from the implementation of HTS techniques in addition to commonly used quantitative molecular techniques. The potential advantage of HTS techniques is their unbiased targeting and output, open to genome- and transcriptome-wide investigation. Specifically, when investigating virus–cell systems, experimental output includes both viral and cellular mRNAs and is, therefore, especially suited to comparative analysis and correlation studies between viral transcription and induced modifications in the cell expression profile at population level.

In our present work, we focused on the system formed by B19V and UT7/EpoS1 cells, a commonly used cell line for in vitro studies on the B19V lifecycle. UT7/EpoS1 cells are a subclone of parental UT7/Epo cells, an erythropoietin-committed sublineage originally derived from multipotent UT7 cells [8,9]. Different subclones derived from the parental UT7/Epo may show different degrees of susceptibility and permissiveness to B19V infection [11]. In our UT7/EpoS1 cells, surface molecules such as the glycolipid globoside, CD71 (hTFR1), and the VP1u ligand are uniformly present in the whole-cell population. Globoside, formerly considered the B19V primary receptor [34,35], is now reassigned to a necessary role in intracellular trafficking and permissiveness [36,37]. Meanwhile, hTFR1 (CD71) recently emerged not only as a marker of differentiation towards an erythroid phenotype, but also as a major binding partner with VP1u in the early phases of attachment and penetration into cells [38,39]. On these grounds, assuming uniform susceptibility to the first steps of the viral replicative cycle, heterogeneity at the intracellular level needs to be hypothesized to account for restriction to B19V, since only a small percentage of cells actually can support productive infection. A limitation of our experimental approach is that, since all information obtained by quantitative molecular techniques or HTS techniques is necessarily averaged over the cell population, it is unable to highlight such single-cell variability.

Within this framework and limits, our investigation yielded information on virus and cell transcriptome modulation in the course of B19V infection in the UT7/EpoS1 cells. mRNAseq analysis of the viral transcriptome returned information in agreement with what was obtained by quantitative molecular methods, including information on differential transcript abundance and post-transcriptional processing [16,17]. The leader sequence from the start of transcription is normally detected at high abundance, and splice boundaries are neatly defined for both the first and second introns, with respective alternative processing patterns. Concerning the left-hand genomic cassette, mRNA1 transcripts, encoding the NS1 protein, are expressed during all stages of infection, at higher relative abundance at early stages and lower relative abundance at late stages. mRNA2 transcripts, which may encode the putative 7.5 kDa and 9 kDa proteins, are highly abundant during all infectious phases, as previously known. According to the string analysis, the first proximal polyadenylation site pAp1 is more easily detected than the second proximal site pAp2, which does not emerge against the background. Concerning the right-hand genomic cassette encoding the VP and 11 kDa proteins, the increase at late compared with early times is less evident, and usage of the pAd cleavage–polyadenylation site is less defined. More accurate mapping was likely prevented by the sequencing strategy employed, which did not yield a uniform coverage across this distal region of mature transcripts.

mRNAseq analysis of the cellular transcriptome compared variation occurring in a time course of infection, either with respect to a basal, not-infected state for single time points or with respect to subsequent different time points. By this approach, the essentially diachronic information obtained encompassed the dynamics and time-dependent variation in the cellular expression profile of the virus-exposed cell population, matching the time-dependent analysis of the viral expression profile. The introduction of the virus in the system strongly differentiated the infected cells from the basal state already at 2 hpi, and increasingly at later time points, indicating the virus as a key driver of variation. Within a very complex interaction network, downregulation of genes involved in the immune/cytokine/interleukin response was prominent from earlier time points throughout the time course of infection (Appendix A, Table A3: 2 hpi, cluster #1; 16 hpi, cluster #1; and 48 hpi, clusters #1 and #2). Conversely, upregulation of genes involved in cell stress response was found at 2 hpi (cluster #2), and genes involved in cell cycle regulation were modulated at 16 hpi (cluster #2) and 48 hpi (cluster #3) (see also Supplemental File S5 for cell cycle analysis). The observed variation at the population level was the result of multiple individual, and possibly heterogeneous, single-cell trajectories, incorporating both the virus-induced and, to some extent, the intrinsic evolving dynamics of the cell population. As a working hypothesis, the early response is likely distributed over the whole-cell population, and its outcome may contribute to the definition of the restrictive characteristics of this cell population. The late response, meanwhile, not necessarily confined to the productively infected subset of cells, may shape the cellular environment to determine the outcome of infection. Single-cell mRNA seq analysis will necessarily be required to better dissect the whole spectrum of viral-induced modulation of the cellular environment.

The present dual-transcriptome analysis conducted on UT7/EpoS1 cells can be compared with a recent analysis carried out on differentiated EPCs, which constitute the cellular system more closely representative of the natural target cells in bone marrow [12]. The viral transcriptome showed quite comparable profiles across the time points, thus corroborating the validity of UT7/EpoS1 cells as a model system supporting B19V replication. Meanwhile, concerning the cellular transcriptional landscape, the differences in the two systems were prevalent, such that some comparative information could be obtained only by including in the analytical model all variables: cell type, infection status, and time point post-infection. Given the interaction network, UT7/EpoS1 cells show a relative upregulation of genes involved in cell cycle regulation (Appendix A, Table A4: 2 hpi, cluster #1, and 48 hpi, cluster #1), and a relative underexpression of genes involved in cytokine and interleukin signaling (e.g., 16 hpi, cluster #2, and 48 hpi, clusters #3 and #5). Differences between the two systems, thus, involve key cellular processes, where the impact of the virus may differ substantially; thus, caution should be exercised when extrapolating the results obtained in the UT7/EpoS1 cells to the natural target EPCs.

## 5. Conclusions

In our present work, a characterization of both viral and cellular expression profiles in the course of B19V infection of UT7/EpoS1 was obtained, reconstructing the viral transcriptome and highlighting variations in the cellular transcriptome landscape. A comprehensive analysis of the modulation of the expression profile in a model cell population was presented, also providing a comparison with the in vitro-differentiated EPCs, which are most closely representative of the target erythroid cells in bone marrow. Similarities and differences in the two systems emerged, thus posing a note of caution when extrapolating experimental data obtained in UT7/EpoS1 to EPCs. Furthermore, compared with bulk techniques, a single-cell analysis approach, now more easily attainable, will better elucidate the complex virus–cell relationship and the impact of viral infection on target cells [40]. In turn, this will allow a better comprehension of the pathogenic mechanisms of infection and a better definition of potential antiviral strategies.

## Figures and Tables

**Table 1:** Fractional distribution of HTS RNA reads on B19V transcription map.

Region	nt Start *	nt End *	%16 hpi ^§^	%48 hpi ^§^
Leader	530	585	0.23	0.28
Intron NS	586	2088	0.06	0.02
Exon Long	2089	2208	0.24	0.24
Exon Short	2209	2362	0.17	0.19
pAp1	2363	2841	0.08	0.07
pAp2	2842	3141	0.05	0.04
Exon VP1	2842	3223	0.07	0.07
Exon VP2	3224	4882	0.04	0.03
pAd	4883	5189	0.05	0.05
Terminal	5190	5213	0.03	0.01

* nt start and nt end indicate the genomic regions selected for HTS count; ^§^ percentage of reads mapped to the different genomic regions, normalized to the respective length.

**Table 2:** Frequency of alternative mRNA-processing events at the indicated sites.

Region	nt Start *	nt End *	%16 hpi ^§^	%48 hpi ^§^
**Splicing**				
D1-no splicing	585	586	0.13	0.05
D1-A1.1	586	2088	0.51	0.53
D1-A1.2	586	2208	0.37	0.42
D2-no splicing	2362	2363	0.41	0.41
D2-A2.1	2362	3141	0.25	0.21
D2-A2.2	2362	4882	0.34	0.38
**Cleavage**				
pAp1	2841	2842	0.61	0.51
pAp2	3141	3142	N.D.	N.D.
pAd	5190	5191	0.37	0.71

* nt start and nt end indicate the genomic positions selected for HTS count; ^§^ percentage of reads mapped to the different genomic regions, normalized to the respective alternative processing patterns. N.D.: not determined.

## Data Availability

The original raw FASTQ reads presented in this study have been submitted to the European Nucleotide Archive (accession: PRJEB107099).

## Supplementary Materials

The following supporting information can be downloaded at https://www.mdpi.com/article/10.3390/v18090988/s1. Supplemental File S1: DGS analysis in UT7/EpoS1; Supplemental File S2: GSEA in UT7/EpoS1; Supplemental File S3: STRING analysis in UT7/EpoS1; Supplemental File S4: STRING analysis of UT7/EpoS1 vs. EPCs; Supplemental File S5: Cell cycle analysis in B19V mock-infected and infected UT7/EpoS1 cells.

## Abbreviations

The following abbreviations are used in this manuscript:

B19V	Parvovirus B19
PBMC	Peripheral Blood Mononuclear Cell
EPCs	Erythroid Progenitor Cells
HTS	High-Throughput Sequencing

## Appendix A

**Table A1:** Primer pairs used for qPCR and qRT-PCR analyses of B19V targets.

**Primer**	**Sense**	**Primer**	**Antisense**	**DNA Target**
18Sfor	CGGACAGGATTGACAGATTG	18Srev	TGCCAGAGTCTCGTTCGTTA	Genomic 18S rDNA
R2210	CGCCTGGAACACTGAAACCC	R2355	GAAACTGGTCTGCCAAAGGT	Virus DNA
**Primer**	**Sense**	**Primer**	**Antisense**	**RNA Target**
R1882	GCGGGAACACTACAACAACT	R2033	GTCCCAGCTTTGTGCATTAC	mRNA1
R2210	CGCCTGGAACACTGAAACCC	R2355	GAAACTGGTCTGCCAAAGGT	mRNA1–5, central exon
R4899	ACACCACAGGCATGGATACG	R5014	TGGGCGTTTAGTTACGCATC	mRNA3–5, distal exon

**Table A2:** Sequence strings used for selection and mapping of mRNAseq reads: strings containing the specific sequence as derived from processing of pre-mRNA at the indicated sites.

Region	nt Start *	nt End *	Sequence
**Splicing**			
D1-no splicing	585	586	GTGAGCTAACTAACAGGTATTTATACTACTTG
D1-A1.1	586	2088	GTGAGCTAACTAACAGATGCCCTCCACCCAGA
D1-A1.2	586	2208	GTGAGCTAACTAACAGGCGCCTGGAACACTGA
D2-no splicing	2362	2363	ACCAGTTTCGTGAACTGTTAGTTGGGGTTGAT
D2-A2.1	2362	3141	ACCAGTTTCGTGAACTGTGCAGCTGCCCCTGT
D2-A2.2	2362	4882	ACCAGTTTCGTGAACTCTACAGATGCAAAACA
**Cleavage**			
pAp1	2841	2842	TTGCTCGTATTAAAAATAACCTTAAAAACTCT TAACCTTAAAAACTCTCCAGACTTATATAGTC CCAGACTTATATAGTCATCATTTTCAAAGTCA
pAp2	3141	3142	TGGGAATAAATCCATATACTCATTGGACTGTA TACTCATTGGACTGTAGCAGATGAAGAGCTTT GCAGATGAAGAGCTTTTAAAAAATATAAAAAA
pAd	5190	5191	AAAATTTAGAAAAATAAACATTTGTTGTGGTT AACATTTGTTGTGGTTAAAAAATTATGTTGTT AAAAAATTATGTTGTTGCGCTTTAAAAATTTA

* nt start and nt end indicate the genomic positions selected for HTS count.

**Table A3:** Interaction network analysis in UT7/Epos1 cells via STRING.

Cluster Numb.		Gene Count	Cluster Coeff.	Protein Names	Reactome Pathways	Sum LogFC	Avg Log FC
2 hpi	1	18	0.78	PTGS2, CCL2, SLAMF7, IL1B, CMKLR1, NFKBIZ, CISH, CD47, CD69, FCGR2B, F2R, LIF, ARG1, CCL4, CSF1, CCR4, CCRL2, IL10RB	Immune system Signaling by interleukins	−23.96	−1.33
	2	12	0.82	PPP1R15A, HERPUD1, TSC1, DNAJB9, CHAC1, ASNS, XBP1, HSPA5, ATF4, HYOU1, ID2, GABARAPL1	Cellular responses to stress	18.02	1.50
	3	6	0.84	CEBPB, KLF6, FOS, CCND1, H2BC3, DUSP2	Generic transcription pathway	−3.42	−0.57
16 hpi	1	33	0.67	GADD45B, KLF10, IFRD1, IER3, BTG2, NR4A1, TNFAIP3, FOSB, FOS, DDIT3, NFKBIA, JUNB, JUN, CDKN1A, ATF3, GADD45A, SERPINE1, PELI1, THBS1, MAP3K8, BIRC3, PTGS2, INHBA, AREG, PRDM1, PTHLH, H2BC3, MAFF, TRIB1, DDB2, PPM1D, DUSP6, TRIB3	Signal transduction Generic transcription pathway Cytokine signaling	−67.15	−2.03
	2	18	0.75	CCL2, CXCL8, TNFSF10, CD69, FAS, CCL4, CSF1, IRF1, CXCL2, IFIH1, IL4R, CD274, LIF, CXCR3, GBP2, GBP4, IL6ST, RNF213	Immune system Signal transduction	−29.15	−1.62
	3	14	0.92	KIF20A, AKAP12, ESPL1, PIF1, RRM2, CDCA3, CCNF, KIF18B, NEK2, CENPE, INCENP, BUB1, PLK1, CEP250	Cell cycle	10.66	0.76
	4	6	0.84	HIF1A, EGLN3, PDK1, P4HA1, SLC16A3, PPFIA4	--	5.60	0.93
	5	4	0.83	GFPT2, SAT1, HK2, MPI	--	−0.88	−0.22
48 hpi	1	30	0.77	PRF1, IL7R, CD276, CD69, GZMB, CD63, SERPINE1, SCARB1, CD36, CCRL2, CD274, CSF1, TNFRSF9, CCL4, CCL2, CXCL2, CXCL8, IL4R, IKZF2, TNFSF9, CXCR3, CCR7, SRGN, AGER, P2RX7, CMKLR1, IL1RL1, CCR4, MARCHF8, GBP4	Immune system Signal transduction	−46.39	−1.55
	2	20	0.82	CDC25A, CDC6, CDC20, TRIP13, GINS4, NCAPD2, DEPDC1, CCNF, TACC3, CENPN, ESPL1, BUB1, PLK1, TOP2A, TIPIN, GINS3, NUP107, STAG1, EZH1, TCF19	Cell cycle	−1.00	−0.05
	3	14	0.77	PIGQ, GPI, ENO2, PFKL, ALDOA, ENO3, GALK1, PHGDH, UAP1, BCKDHA, PCK1, PKLR, IDH3A, ALDOC	Metabolism of carbohydrates	14.89	1.06
	4	10	0.85	BAG1, TOMM40, DNAJA1, HSPA9, HSPE1, TIMM8B, TIMM17A, DNAJA4, TIMM10, UBE2J1	Mitochondrial protein import	−7.94	−0.79
	5	9	0.92	PSMC4, PSME3, PSMA3, PSMD14, PSMD12, ADRM1, PSMC2, UBE2N, AQP3	FCERI-mediated NF-kB activation	−10.15	−1.13
	6	8	0.89	POP4, NOP2, NOP56, RRP9, SDAD1, DDX21, NOLC1, PNO1	Metabolism of RNA	−9.66	−1.21
	7	7	0.85	SLC2A3, BNIP3, HIF1A, P4HA1, NDRG1, STC1, PPFIA4	--	2.47	0.35
	8	7	0.88	ABCE1, EIF2S1, ETF1, EIF5, EIF3J, ABCF2, ANKZF1	Translation	−6.01	−0.86
	9	7	0.86	ATF3, NFKBIZ, KLF6, JUNB, NR4A1, MAFF, ERRFI1	--	−13.12	−1.87
	10	6	0.84	TGM2, COL2A1, SPP1, THBS3, ITGA9, ITGA5	Integrin cell surface interactions	2.57	0.43
	11	6	0.81	PCNA, RAD51C, FEN1, PAN2, UNG, ZMIZ1	DNA repair	−0.99	−0.16
	12	6	0.78	SELENBP1, TNS1, EPB42, ADD2, ADD3, AKAP12	--	4.68	0.78
2-48 hpi	1	16	0.92	CDC25A, CDC6, PIF1, DEPDC1, CCNG1, KIF20A, NEK2, KIF23, ZWINT, TOP2A, PLK1, BUB1, FEN1, ESPL1, RPS6KA3, MAST4	Cell cycle	5.38	0.34
	2	13	0.76	PPP1R15A, MAFF, TNFAIP3, FOS, DUSP1, CEBPG, ID1, EGR3, BTG2, NR4A1, PDCD4, MAP3K1, HOMER1	--	−8.60	−0.66
	3	13	0.79	PMAIP1, HSP90B1, CALR, XBP1, HSPA5, DNAJB1, SEC61A1, GMPPB, TGM2, DNAJC12, DNAJA1, SDF2L1, HSPH1	Cellular responses to stress	−15.27	−1.17
	4	9	0.81	HSD17B10, ACADS, HMGCL, ALDH6A1, EHHADH, MLYCD, ALDH8A1, MCEE, SYNGR1	Metabolism	4.64	0.52
	5	8	0.96	PSMC4, EGLN3, PSME3, PSMD14, PSMD12, ADRM1, PSMC3, PSMC2	Proteasome assembly	−7.19	−0.90
	6	8	0.87	ENO2, PYGB, ALDOC, PCK1, GLUL, PFKM, GPI, GPD1	Metabolism	6.03	0.75
	7	7	0.81	SNAI2, TGFB3, SERPINE1, SMAD3, TGIF2, LTBP1, ZMIZ1	Signal transduction	2.39	0.34
	8	6	0.81	NDRG1, LDHA, PDK1, P4HA1, SLC1A5, SLC16A3	Pyruvate metabolism	6.31	1.05
	9	6	0.86	RRP12, RRP9, LHPP, DDX21, MYBBP1A, PNO1	rRNA processing	−4.88	−0.81
	10	5	0.87	SLC25A1, D2HGDH, IDH1, IDH2, GLRX	Metabolism	5.67	1.13
	11	5	0.60	GAA, GLA, NPC1, IDS, HES1	--	−0.86	−0.17
	12	5	0.60	ATF3, DDIT3, DBP, ERRFI1, IFRD1	--	−6.52	0.34

**Table A4:** Interaction network analysis in UT7/Epos1 vs. EPC via STRING.

Cluster Numb.		Gene Count	Cluster Coeff.	Protein Names	Reactome Pathways	Sum LogFC	Avg Log FC
2 hpi	1	15	0.88	MYC, FBXO5, E2F1, HBEGF, MT2A, EIF4A2, CDKN2C, CCND3, E2F3, KLF5, NOTCH1, CDR2, LBR, HDGF, NFE2	Mitotic G1 phase and G1/S transition	5.20	0.35
	2	10	0.80	ELL2, POLR2K, POLR2A, ELOA, CCNT1, GTF2A2, TAF13, H2BC12, H4C3, ARID4A	Transcription by RNA polymerase II	1.64	0.16
16 hpi	1	10	0.87	PTGS2, FOS, NFKBIZ, BTG2, ATF3, FOSB, NR4A1, MT2A, JUNB, EGR3	Transcription regulator activity	−24.89	−2.49
	2	8	0.84	CD274, CXCL2, CD69, CCL4, CSF1, CCL2, IL1R2, IL3RA	Response to cytokine	−31.83	−3.98
	3	6	0.81	IRF1, EPSTI1, IFI27, GBP4, GBP2, RNF213	Interferon signaling	−6.70	−1.12
	4	6	0.93	PKM, ENO3, ENO2, HK2, PFKP, CALB2	Glycolysis	9.64	1.61
	5	6	0.88	HIF1A, EGLN3, PDK1, P4HA1, EFNA3, HIPK2	Cellular response to hypoxia	2.86	0.48
48 hpi	1	22	0.85	BUB1, ESPL1, PLK1, CENPE, MKI67, PRC1, NEK2, CENPF, TUBG1, CCNF, PLK3, TPX2, GINS2, RACGAP1, KIF23, KIF2C, KIF20A, KIF15, DEPDC1, INCENP, KIF5A, RHOT1	Cell cycle; mitotic	18.68	0.85
	2	14	0.77	CD63, SCARB1, ITGB4, TGM2, ITGA9, LAMC1, ITGB1, ITGA4, LAMB3, TIMP3, MERTK, LTBP1, DMD, JAM3	Extracellular matrix organization	−2.48	−0.18
	3	13	0.77	CD276, CD69, CST7, TNFRSF9, IL18RAP, IL1R2, CCL4, CCL5, CXCL3, CD83, CCR4, TNFSF9, MARCHF1	Cytokine signaling	−39.82	−3.06
	4	13	0.84	ISG15, IFIH1, IRF2, SAMD9L, IFI27, IFI44L, XAF1, IRF9, ISG20, RNASEL, RNF213, SAMHD1, APOL6	Interferon alpha/beta signaling	0.95	0.07
	5	10	0.66	CSF3R, IL27RA, CSF2RA, IL3RA, LIF, IL13RA1, IL4R, JAK2, IL15RA, IL9R	Interleukin signaling	−17.54	−1.75
	6	10	0.81	PCNA, POLE4, POLL, RAD51C, BARD1, AARS1, NUDT15, PNPT1, POLH, POLB	DNA repair	−4.98	−0.50
	7	9	0.83	SLC2A3, ENO2, PFKL, ALDOA, PDK1, PMM1, ENO3, ALDOC, CALB2	Glycolysis	10.08	1.12
	8	9	0.76	UQCRQ, NDUFS2, NDUFC2, ATP5PF, NDUFB2, TIMM17A, TIMM8B, MGST3, ATP6V1G1	Respiratory electron transport	−5.21	−0.58
	9	7	0.92	SERPINE1, EGF, FURIN, DAB2, LDLR, STAM, SH3GL2	Clathrin-mediated endocytosis	−8.65	−1.24
	10	7	0.79	MAFF, GCLC, ODC1, CTH, MTHFD2, SLC7A11, GFPT1	Ferroptosis	4.88	0.70
	11	6	0.82	PPARG, CREBBP, SP1, PML, AGO4, HDAC9	TGF-beta signaling pathway	−2.45	−0.41
	12	6	0.84	IDH2, IDH1, BCKDHA, IDH3A, ALDH6A1, CRAT	TCA cycle	6.68	1.11
