# Supplementary material for: Parvovirus B19 and Cellular Transcriptome Dynamics in UT7/EpoS1 Cells
Source: Viruses. 2026 Sep 8;18(9):988. doi: 10.3390/v18090988 (PMC13612003; doi:10.3390/v18090988)
Supplement: Supplementary file 1 [file viruses-18-00988-s001.zip › Supplemental File S5.pdf]

UT7/EpoS1 cells, mock-infected

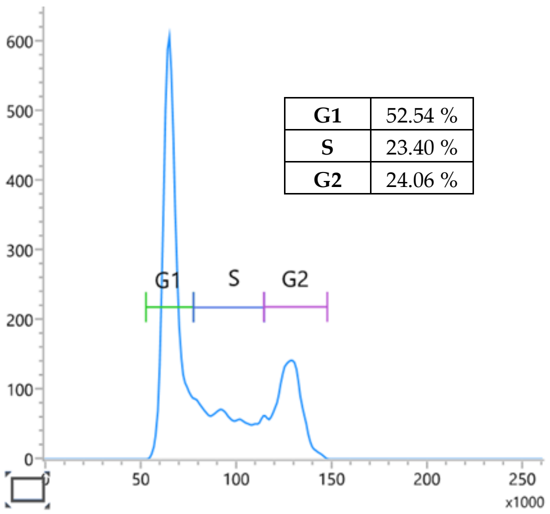

UT7/EpoS1 cells, B19V infected

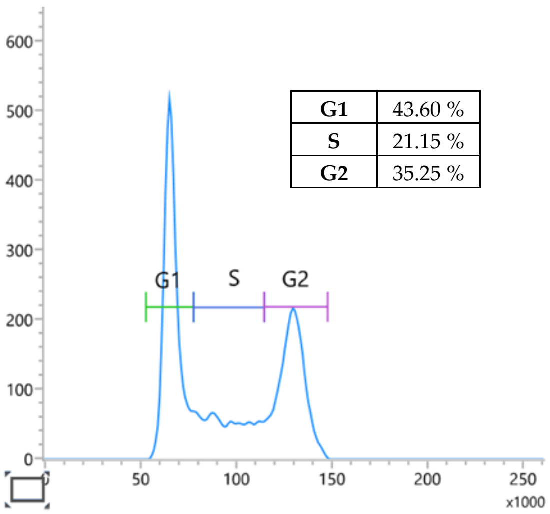

Cell cycle analysis in B19V mock infected and infected UT7/EpoS1 cells. UT7/EpoS1 cells were collected at 48 hpi, fixed in 70% ethanol, washed, and then resuspended in FxCycle™ PI (Propidium Iodide)/RNase stain (Molecular Probes) for 30 minutes at room temperature. Flow cytometry acquisition was performed on the BD FACSLyric (BD Biosciences) using a 488 nm blue laser with a 586/42-nm bandpass filter at a low flow rate for optimal resolution. Histograms generated with BD FACSuite software (BD Biosciences) show DNA content distribution across cell cycle phases. (Propidium iodide intensity, X-Axis; Counts, Y-Axis). The inset tables contain a quantitative (percentage) assessment of cell cycle distribution.
